# Dietary inclusion of Peptiva, a peptide-based feed additive, can accelerate the maturation of the fecal bacterial microbiome in weaned pigs

**DOI:** 10.1186/s12917-020-02282-x

**Published:** 2020-02-18

**Authors:** Prakash Poudel, Crystal L. Levesque, Ryan Samuel, Benoit St-Pierre

**Affiliations:** Department of Animal Science, South Dakota State University, Animal Science Complex, Box 2170, Brookings, SD 57007 USA

**Keywords:** Weaning, Pig, Swine, Peptide, Gut, Microbiome, Bacteria

## Abstract

**Background:**

Weaning is one of the most critical transition stages of the swine production cycle, as the piglet gut physiology and microbiome need to rapidly adapt to changes in diet and environmental conditions. Based on their potential for producing a vast array of bioactive molecules, peptide formulations represent a largely untapped source of compounds that could be developed into feed additives to benefit animal health and nutrition. In this context, a commercial-scale nursery trial was performed to evaluate the impact of low inclusion of a peptide-based feed additive (Peptiva, Vitech Bio-Chem Corporation) on the performance and fecal microbiome of weaned pigs.

**Results:**

While no significant differences in body weight, daily gain, daily feed intake nor gain:feed were observed between control and treatment animals (*P* > 0.05), an effect of Peptiva on the fecal bacterial composition of weaned pigs was observed. The first main observation was that the fecal bacterial profiles from pigs fed Control-Phase II and Control Phase III diets were found to be very distinct, suggesting that a transition or succession stage had occurred between the two phases. *Lactobacilli*, represented by four main OTUs (Ssd-00002, Ssd-00019, Ssd-00025, and Ssd-00053), were more abundant at the end of Phase II (*P* < 0.05), while *Streptococci*, mostly represented by OTUs Ssd-00039 and Ssd-00048, were in higher abundance at the end of Phase III (*P* < 0.05). Secondly, the fecal bacterial composition from pigs fed Peptiva Phase II diets showed similarities to both Control-Phase II and Control Phase III samples, while there was no difference in fecal bacterial composition between Control-Phase III and Peptiva Phase III samples. For instance, OTUs Ssd-00019,and Ssd-00053 were in lower abundance in Peptiva Phase II samples compared to Control Phase II (*P* < 0.05), but no significant difference was observed in the abundance of these two OTUs when comparing Peptiva Phase II to Control Phase III (*P* > 0.05).

**Conclusions:**

Together, these results suggest that Peptiva can modulate the composition of the swine microbiome during a specific window of the nursery stage, potentially by accelerating its maturation.

## Background

Weaning is one of the most critical transition stages of the swine production cycle, as decreased feed intake and poor performance from sudden changes in diet and environment can result in severe economic losses [1, 2]. While a number of physiological conditions contribute to the performance and health challenges that commonly occur during the nursery phase, gastrointestinal dysfunction is generally involved. Typically, a combination of prolonged intestinal inflammation, immature immune system and transitioning gut microbial communities result in a compromised gut epithelial lining, decreased nutrient digestibility, and increased susceptibility to pathogen infection [1, 3–11]. Together, these conditions can lead to a higher incidence of diarrhea, resulting in higher weaned pig morbidity and mortality.

Conventional approaches to reduce the impact of weaning on nursery pig health and performance have typically combined antibiotic use to reduce the pathogen load with inclusion of high-quality protein ingredients to facilitate digestion and absorption [12]. However, implementation of stricter regulations on the prophylactic use of medically important antimicrobials, as well as higher costs of traditionally used protein sources such as fish meal, have created a need for effective substitutes and the development of innovative strategies. For instance, products such as essential oils and antimicrobial peptides are becoming more widely used as alternative antimicrobials, while modified plant ingredients with reduced levels of anti-nutritional factors (e.g. enzymatically or microbially modified soybean meal) are being included as lower cost protein-rich sources in dietary formulations [13–15]. In addition to these substitutes, feed additives are also developed to target other functions, such as enhancing the immune response of weaned pigs (e.g. immunoglobulin or omega-3 fatty acids), stimulating digestive functions (e.g. butyrate, glutamate, threonine or cysteine), or promoting the establishment of beneficial gut microorganisms (probiotics, prebiotics) [16–18].

Amongst the various products available, peptides have the unique potential to be used as multipurpose feed additives. Indeed, they are cost effective means of providing amino acids, as they are more stable, soluble, and can be absorbed at a faster rate than free amino acids [19–21]. In addition, certain types of peptides can control various physiological functions by acting as either antimicrobials, antioxidants, immuno-modulators or signaling molecules [20, 22, 23]. In the case of bioactive peptides supplemented in feed, they may act on either host cells and / or on the host’s microbiome [24–26]. As an example of peptide signaling to host cells, exorphins have been shown to modulate gastrointestinal motility, secretions, and endocrine metabolism once they have been released by digestion and absorbed by the gut epithelium [27]. Conversely, modulation of gut microbiome composition by certain antimicrobial peptides has also been reported. For instance, colicins and cecropin AD can help control the proliferation of *Escherichia coli* strains that can cause post weaning diarrhea in swine [25, 28, 29]. Antimicrobial peptides can also have positive effects on performance. Indeed, feeding a combination of lactoferrin, cecropin, defensin and plectasin resulted in higher average daily gain and final body weight compared to unsupplemented diets [30]. Similarly, apparent total tract digestibility of either dry matter or crude protein was found to be higher with dietary supplementation of the antimicrobial peptide-P5 [31].

Considering the importance of beneficial gut microbial communities for animal health and nutrition, manipulating the gut microbiome using peptides would represent an additional tool towards improving resistance to pathogens, optimizing the use of alternative feed ingredients or providing other benefits to the host animal. Typically, bioactive peptides remain inactive until they are released from their parent protein as a result of chemical, enzymatic, or microbial hydrolysis [32]. Since their functional characteristics would depend on their length as well as their amino acid composition and sequence [20, 23], there likely exists a wide range of potential bioactive peptides that have yet to be identified or characterized. Indeed, the search for novel bioactive peptides is still ongoing even for highly investigated sources such as milk [33]. Thus, a reasonable expectation would then be that many peptide formulations would contain bioactive peptides that can perform functions other than simply supplying dietary amino acids. However, as the effects of peptide feed additives on the gut microbiome of food animals remain largely unexplored, additional insight is required to develop further improvements in this field.

In this context, the aim of the study presented in this report was to determine the effect of a commercially-formulated peptide additive, Peptiva, on the performance and fecal bacterial community profiles of weaned pigs raised in a commercial wean-to-finish swine facility. This product has been previously reported as an acceptable protein supplement in nursery diets [34], but had not been tested at low inclusion levels. In the current study, Peptiva supplementation did not result in improved weight gains or feed efficiency of weaned pigs under the conditions tested, but it was found to affect the fecal microbiome composition of animals during the first few weeks after weaning.

## Results

### Effect of low inclusion of Peptiva on swine performance during the nursery phase

To test the ability of Peptiva to improve the availability of dietary amino acids in swine nursery phase diets, animals fed a Peptiva-supplemented diet that included only 90% of the recommended amino acids requirements for nursery phase diets (PEP10) were compared to animals fed the control diet (CON). After the first 3 weeks, no effect of diet on body weight was observed (*P* > 0.05; Table 1). Starting at week 4, however, pigs fed the CON diet tended to be heavier than PEP10-fed pigs (*P* = 0.07), with CON-fed pigs continuing to be heavier than PEP10-fed pigs through to week 6 (*P* < 0.05). While there was no difference in average daily feed intake during Phases I and II across dietary treatments, an effect of diet on daily feed intake during Phase III was observed, where CON-fed pigs had greater daily intake than PEP-fed pigs (*P* < 0.05). No effect of dietary treatment on average daily weight gain or gain:feed was observed. While there were 3% fewer pigs removed from the PEP10 group compared to the CON group for the entire trial period (6 wks), a statistical difference in net pig removal rate by diet was not detected. No significant differences were noted in pen weight variation amongst treatment groups.

**Table 1:** Growth performance of weaned pigs fed diets containing Peptiva formulated at 100 (PEP) or 90% (PEP-10) of amino acid requirements (NRC (2012)^1^

	Control	PEP	PEP-10	SEM	***P***-value
Body weight, kg					
d0	5.9	5.8	5.7	0.1	0.602
d6	6.2	6.2	6.2	0.2	0.948
d13	7.8	7.7	7.6	0.1	0.267
d20	10.4	10.2	9.9	0.3	0.396
d27	12.6^a^	12.4^ab^	12.1^b^	0.2	0.067
d34	16.2^a^	15.8^ab^	15.2^b^	0.2	0.011
d41	20.8^a^	20.5^ab^	19.4^b^	0.3	0.008
Average daily gain, kg/d					
d0 – d7	0.063	0.054	0.062	0.024	0.958
d8 – d21	0.256	0.253	0.234	0.014	0.476
d22 – d42	0.471	0.469	0.440	0.019	0.415
Average daily feed intake, kg/d					
d0 – d7	0.108	0.096	0.097	0.014	0.797
d8 – d21	0.309	0.300	0.294	0.022	0.892
d22 – d42	0.659^a^	0.614^b^	0.633^ab^	0.012	0.034
Gain:feed, kg:kg					
d0 – d7	0.54	0.52	0.60	0.16	0.928
d8 – d21	0.81	0.83	0.76	0.05	0.528
d22 – d42	0.71	0.78	0.70	0.03	0.180
Pigs removed, #/pen	2.4	1.9	2.0	0.42	0.700
Total removed, #	36	35	26		
Total started, #	360	383	360		
Removal, %	10	9.1	7.2		
Pen coefficient of variation					
d0	0.230	0.199	0.226	0.011	0.083
d21	0.248	0.247	0.249	0.021	0.999
d42	0.240	0.242	0.269	0.021	0.545

^1^Experimental diets were provided as part of a 3-phase nursery pig feeding program (Phase 1: 0-7d; Phase 2: 8-21d; Phase 3: 22-42d). Peptiva was included at 1.0, 0.5, and 0.3%, respectively). A total of 46 pens of 24 pigs/pen were included in the analysis (n = 16, 15, and 15 pens for CON, PEP, and PEP-10, respectively)

Means in the same row with different superscripts are significantly different as determined by the Tukey honest significant difference test

### Effect of diet composition and Peptiva supplementation on the fecal bacterial profile of weaned pigs

To investigate the potential of Peptiva as a modulator of gut microbiome composition in weaned pigs, a comparative analysis using fecal bacterial communities as a proxy was performed on samples collected at the end of Phase II and at the end of Phase III. The average number of high-quality, non-chimeric reads for 16S rRNA gene sequences across the four sample sets (CON II, CON III, PEP II and PEP III) ranged from 14,972 ± 2792 to 26,020 ± 3191 (Table 2), with numerical differences amongst means not found to be significant (*P* = 0.16). Firmicutes was the most highly represented phylum, with sample set averages ranging from 77.4 to 85.3% (Table 3; Supplementary File 3). While these variations in abundance at the phylum level were found to be only numerical, the differences in representation for two families belonging to Firmicutes were found to be significant (*P* < 0.05). *Lactobacillaceae* were more abundant in CON II samples (44.8%) than in samples from pigs fed the PEP II, CON III or PEP III diets (13.0–16.0%). In contrast, *Streptococcaceae* were in lower abundance in CON II compared to CON III and PEP III (*P* < 0.05). Other well represented families belonging to Firmicutes included *Lachnospiraceae* (5.9–13.2%) and *Clostridiaceae1* (5.9–18.9%), but the observed differences in abundance were not significant (*P* > 0.05). The second most abundant phylum was Bacteriodetes, with *Prevotellaceae* identified as its most highly represented family (11.8% - 16.0); variation across datasets was not found to be significant for either of these taxonomic groups.

**Table 2:** High quality sequence read yield from each sample set

Sample set	Sequence yield	Sequence quality range^**#**^
**CON II**	22,541 ± 4421	37.0–37.8
**CON III**	26,020 ± 3191	37.3–37.9
**PEP II**	14,972 ± 2792	37.3–37.8
**PEP III**	18,465 ± 3595	37.2–37.8

^#^Range of mean Phred quality scores per sample for each sample set

**Table 3:** Relative abundance (%) of the main bacterial taxonomic groups in representative fecal samples from Control and Peptiva-fed pigs in each of Phase II and III diets

Taxonomic affiliation	Con PII	Pep PII	Con PIII	Pep PIII
**Firmicutes**	81.3 ± 6.1	77.4 ± 7.7	81.7 ± 6.6	85.3 ± 6.8
*Lactobacillaceae*^#^	44.8^a^ ± 9.0	13.4^b^ ± 6.8	13.0^b^ ± 5.1	16.0^b^ ± 5.9
*Lachnospiraceae*	13.2 ± 2.9	12.7 ± 2.4	7.8 ± 2.4	5.9 ± 1.3
*Erysipelotrichaceae*	1.5 ± 0.5	3.7 ± 1.5	0.7 ± 0.2	0.7 ± 0.2
*Ruminococcaceae*	5.4 ± 1.6	6.1 ± 2.0	2.2 ± 0.5	2.3 ± 0.7
*Clostridiaceae1*	5.9 ± 3.3	17.3 ± 6.2	11.8 ± 6.6	18.9 ± 6.0
*Peptostreptococcaceae*	1.2 ± 0.4	4.2 ± 2.5	1.1 ± 0.3	2.5 ± 1.0
*Streptococcaceae*^#^	2.1^a^ ± 0.7	9.0^ab^ ± 4.3	32.2^c^ ± 9.2	25.6^bc^ ± 6.6
*Veillonellaceae*	0.5 ± 0.2	1.9 ± 1.3	3.4 ± 1.2	3.1 ± 0.9
unclassified Clostridiales	3.1 ± 1.0	4.2 ± 0.7	2.2 ± 0.4	2.8 ± 0.7
Other Firmicutes	3.6 ± 0.8	4.8 ± 1.1	7.4 ± 1.5	7.4 ± 1.1
**Bacteroidetes**	16.3 ± 5.9	20.4 ± 7.1	17.4 ± 6.5	13.5 ± 6.8
*Prevotellaceae*	14.0 ± 6.1	13.1 ± 6.4	16.0 ± 6.4	11.8 ± 6.8
*Porphyromonadaceae*	1.4 ± 0.6	4.5 ± 2.2	0.5 ± 0.2	1.1 ± 0.8
Other Bacteroidetes	0.8 ± 0.2	2.7 ± 1.8	0.8 ± 0.3	0.6 ± 0.3
**Other Phyla**	1.5 ± 1.0	1.2 ± 0.7	0.3 ± 0.09	0.5 ± 0.3
**Unclassified Bacteria**	0.9 ± 0.2	1.0 ± 0.3	0.6 ± 0.1	0.7 ± 0.2

^#^Taxa showing a significant difference (*P* < 0.05) amongst means of different treatment groups

See Supplementary File 3 for *P* values

Means with different superscripts in the same row are significantly different as determined by the post hoc Nemenyi test for multiple pairwise comparisons

### Comparative analysis of fecal bacterial composition by alpha and beta diversity

**Fig. 1:**
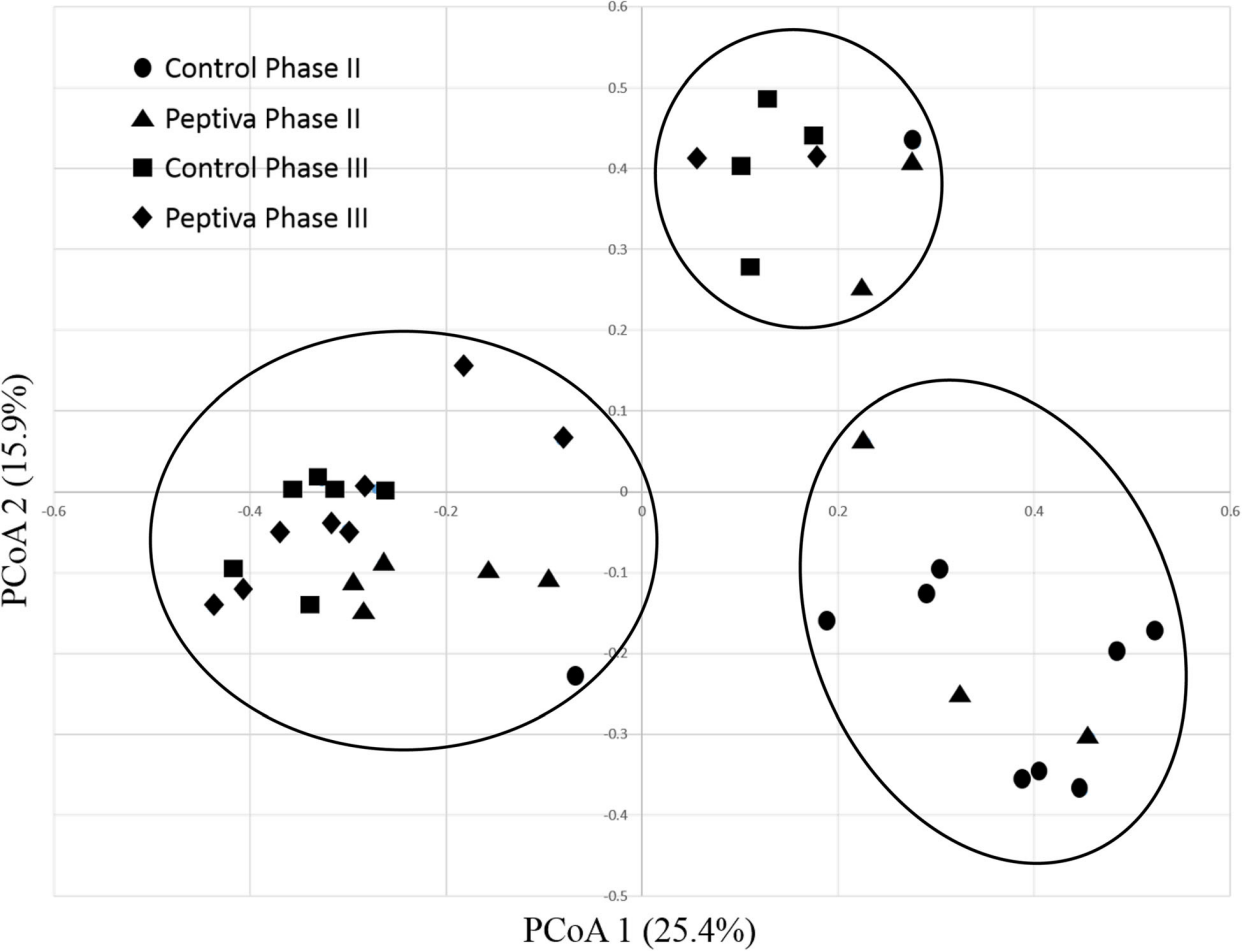
Comparison of fecal bacterial communities from weaned pigs under two different diets at two different time points. Principle Coordinate Analysis (PCoA) was performed using OTU composition-based Bray-Curtis dissimilarity. The x and y axes correspond to Principal Components 1 (PC1) and 2 (PC2), which explained the highest level of variation

Community level compositional differences amongst fecal bacterial communities from CON II, CON III, PEP II and PEP III sample sets were further assessed using alpha and beta diversity analyses. A combined total of 8429 OTUs were identified across all samples analyzed (Supplementary File 2). No statistical difference was observed amongst means of the four dietary treatments for either observed OTUs, Ace, Chao1, Shannon or Simpson indices (*P* > 0.05; Table 4). However, principal coordinate analysis (PCoA) based on Bray-Curtis OTU composition dissimilarity revealed that samples could be clustered into three different groups according to their fecal bacterial community composition (Fig. 1). Furthermore, uneven distribution of samples from different sets amongst the three clusters of the PCoA plot suggested that distinct OTU profiles could be associated with the fecal environments of particular sets of samples.

**Table 4:** Alpha diversity indices and coverage from Control and Peptiva-fed pigs in each of Phase II and III diets

Index	CON II	CON III	PEP II	PEP III	***P***-value
OTUs	383 ± 45	343 ± 32.5	400 ± 48	318 ± 33	0.471
Ace	1395 ± 184	1145 ± 159	1397 ± 187	1110 ± 171	0.510
Chao1	909 ± 121	790 ± 112	920 ± 112	703 ± 86	0.448
Shannon	3.41 ± 0.33	3.12 ± 0.23	3.72 ± 0.27	3.16 ± 0.21	0.357
Simpson	0.197 ± 0.05	0.222 ± 0.03	0.121 ± 0.03	0.189 ± 0.03	0.274
Coverage (%)	92.3 ± 0.93	93.3 ± 0.73	92.1 ± 1.0	93.8 ± 0.77	0.462

### Identification of weaned pig OTUs responding to distinct dietary treatments

**Fig. 2:**
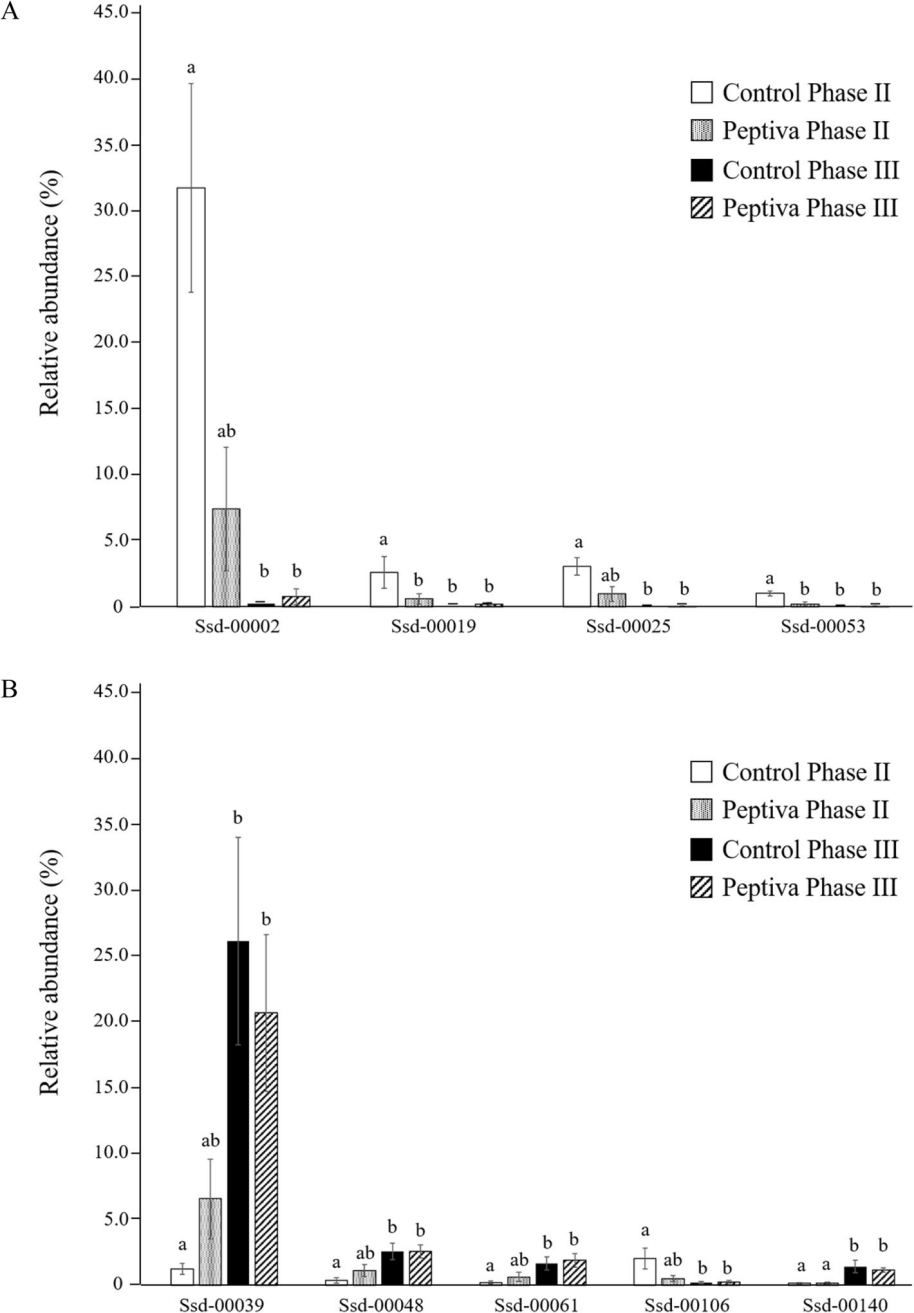
Main bacterial OTUs whose respective abundance was found to vary significantly amongst groups based on the post hoc Nemenyi test for multiple pairwise comparisons (*P* < 0.05). OTUs affiliated to the genus *Lactobacillus* are shown in panel (A) while OTUs affiliated to the genera *Streptococcus* or *Roseburia* are shown in panel (B). For each OTU, means with different superscripts were significantly different as determined by the post hoc Nemenyi test for multiple pairwise comparisons

As the comparative taxonomic composition analysis and PCoA both indicated differences in bacterial composition amongst sample sets, the individual profiles of major OTUs were further investigated. A total of 23 OTUs that were found to have a mean relative abundance of at least 1% in at least one sample set were designated as major OTUs. Of these most abundant OTUs, at least seven were likely to correspond to uncharacterized species, as they each showed less than 95% sequence identity to their respective closest valid taxon. Thirteen major OTUs, all affiliated to Firmicutes, were found to vary across sample types (*P* < 0.05) (Table 5; Supplementary File 3). Pair-wise differences between specific samples for nine of these varying OTUs were further revealed by the post hoc Nemenyi test (Fig. 2). Notably, the respective abundances of OTUs Ssd-00019 and Ssd-00053 were found to be significantly different in CON II compared to PEPII, CON III and PEP III sample sets (*P* < 0.05). OTUs Ssd-00002, Ssd-00025, Ssd-00039, Ssd-00048, Ssd-00061 and Ssd-00106 showed a slightly different profile, with their respective abundances being significantly different between CON II and either CON III or PEP III (*P* < 0.05), while no significant difference was found between PEPII and either CON II, CONIII or PEP III. Also, while Ssd-00140 was found at similar levels in CON II and PEP II, its abundance in these sample sets was significantly lower than in CON III and PEP III (*P* < 0.05).

**Table 5:** Relative abundance (%) of the most abundant OTUs in representative fecal samples from Control and Peptiva-fed pigs in each of Phase II and III diets

OTUs	Con PII	Con PIII	Pep PII	Pep PIII	Closest valid taxon (id%)
**Firmicutes**					
Ssd-00001^a#^	0.1 ± 0.02	10.4 ± 4.9	0.07 ± 0.01	12.0 ± 5.0	*L. amylovorus* (99%)
Ssd-00002^a#^	31.9 ± 7.9	0.3 ± 0.08	7.5 ± 4.7	0.8 ± 0.6	*L. gasseri* (99%)
Ssd-00008^a^	0.06 ± 0.04 0.04	0.05 ± 0.03	1.2 ± 1.1	0.2 ± 0.2	*L. mucosae* (99%)
Ssd-00019^a#^	2.6 ± 1.2	0.1 ± 002	0.6 ± 0.4	0.2 ± 0.1	*L. reuteri* (99%)
Ssd-00025^a#^	3.1 ± 0.7	0.02 ± 0.01	1.0 ± 0.6	0.05 ± 0.03	*L. taiwanensis* (95%)
Ssd-00053^a#^	1.0 ± 0.2	0.02 ± 0.01	0.2 ± 0.1	0.06 ± 0.03	*L. reuteri* (95%)
Ssd-00078^a#^	1.5 ± 0.3	0.01 ± 0.01	1.3 ± 0.8	0.03 ± 0.02	*L. taiwanensis* (88.1%)
Ssd-00013^b#^	0.1 ± 0.08	0.01 ± 0.01	1.0 ± 0.5	0.01 ± 0.01	*S. ventriculi* (98%)
Ssd-00092^b#^	0.2 ± 0.1	0.8 ± 0.4	0.3 ± 0.1	1.0 ± 0.4	*C. paraputrificum* (89%)
Ssd-00238^b^	0.6 ± 0.4	0.5 ± 0.1	1.0 ± 0.3	0.8 ± 0.2	*C. saccharo.* (93%)
Ssd-00134^b^	4.3 ± 2.6	9.1 ± 5.6	13.7 ± 5.5	14.4 ± 4.8	*C. saccharo.* (97%)
Ssd-00014^c^	0.7 ± 0.2	0.6 ± 0.2	3.1 ± 2.1	1.6 ± 0.7	*T. mayombei* (97%)
Ssd-00039^d#^	1.3 ± 0.4	26.2 ± 7.9	6.6 ± 3.0	20.7 ± 6.0	*St. macedonicus* (95%)
Ssd-00048^d#^	0.4 ± 0.2	2.6 ± 0.6	1.1 ± 0.5	2.6 ± 0.5	*St. alactolyticus* (96%)
Ssd-00061^d#^	0.2 ± 0.1	1.7 ± 0.5	0.6 ± 0.4	1.9 ± 0.5	*St. alactolyticus* (90%)
Ssd-00140^d#^	0.2 ± 0.06	1.4 ± 0.5	0.2 ± 0.08	1.2 ± 0.2	*St. salivarius* (91%)
Ssd-00071^e#^	0.1 ± 0.06	1.2 ± 0.6	0.08 ± 0.04	1.1 ± 0.7	*M. indica* (98%)
Ssd-00188^f^	0.5 ± 0.2	1.6 ± 0.9	2.5 ± 1.4	0.6 ± 0.3	*E. rectale* (99%)
Ssd-00106^g#^	2.0 ± 0.8	0.2 ± 0.09	0.5 ± 0.2	0.3 ± 0.1	*R. faecis* (98%)
Ssd-00123^h^	0.2 ± 0.08	0.08 ± 0.04	1.3 ± 0.7	0.03 ± 0.01	Ca. *mitsuokai* (97%)
**Bacteriodetes**					
Ssd-00003^i^	7.0 ± 4.1	9.6 ± 4.4	6.3 ± 3.9	6.5 ± 5.2	*P. copri* (98%)
Ssd-00502^i^	1.4 ± 1.4	0.02 ± 0.01	0.01 ± 0.01	0.01 ± 0.01	*Ma. massiliensis* (84%)
Ssd-00366^j^	1.1 ± 0.5	0.4 ± 0.1	4.0 ± 2.1	0.8 ± 0.7	*Pa. distasonis* (84%)

^**#**^OTUs showing a significant difference (*P* < 0.05) amongst means of different treatment groups

See Supplementary File 3 for *P* values

Taxonomic affiliations: a. *Lactobacillaceae*, b. *Clostridiaceae*, c. *Peptostreptococcaceae*, d. *Streptococcaceae*, e. *Veillonellaceae*, f. *Eubacteriaceae*, g. *Lachnospiraceae*, h. *Erysipelotrichidae*, i. *Prevotellaceae*, j. *Porphyromonadaceae*

Abbreviations: Ca *Catenibacterium*; C *Clostridium;* E *Eubacterium;* L *Lactobacillus*; Ma *Massiliprevotella*; M *Megasphaera*; Pa *Parabacteroides*; P Prevotella; R *Roseburia;* saccharo *saccharoperbutylacetonicum;* S *Sarcina*; St *Streptococcus*; T *Terrisporobacter*

### Associations between main OTUs and dietary treatments

**Fig. 3:**
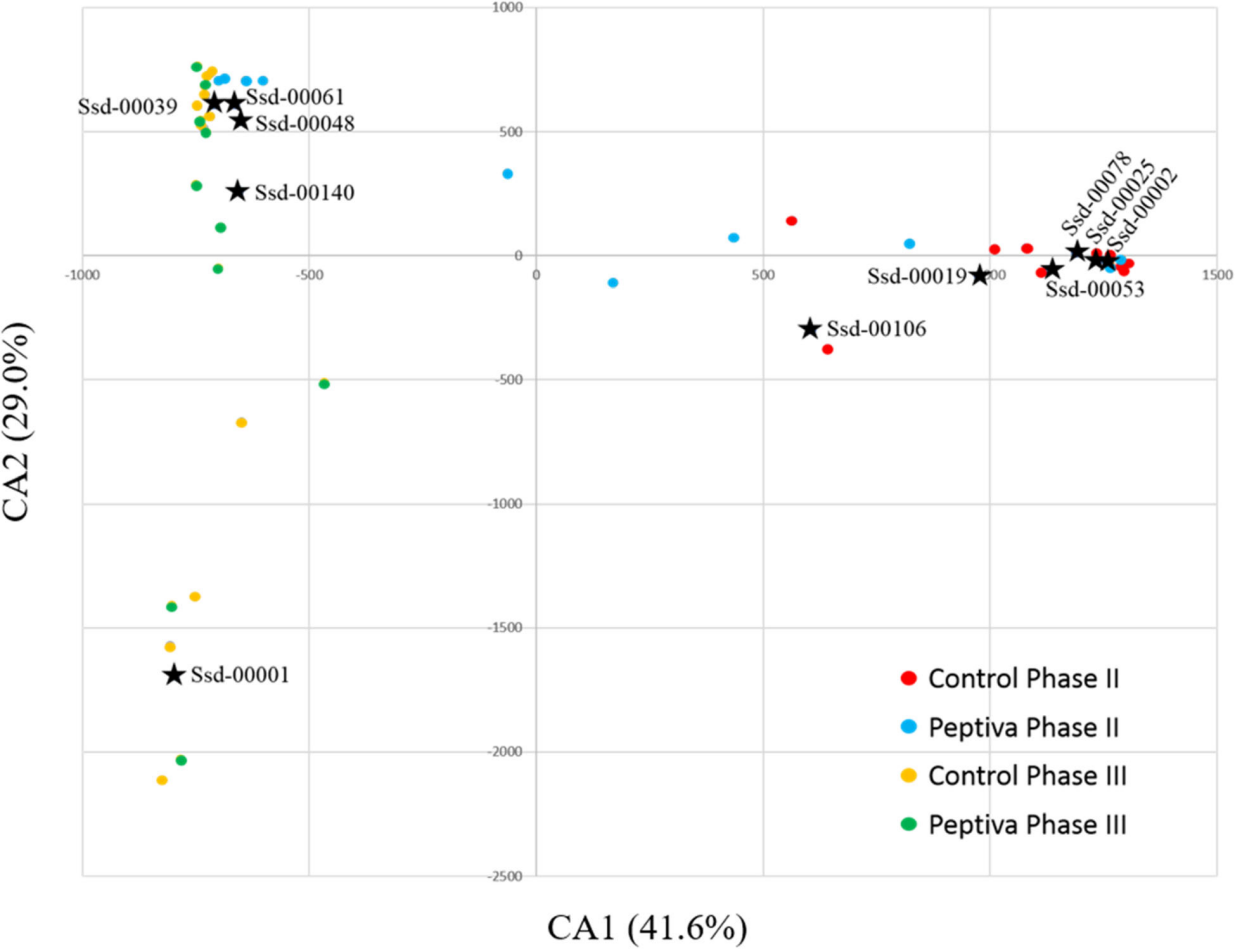
Correspondence analysis (CA) between sample type (circle) and main OTUs (star)

A correspondence analysis was conducted to further explore potential associations between main OTUs and dietary treatments (Fig. 3). All CON II samples clustered together with OTUs Ssd-00002, Ssd-00019, Ssd-00025, Ssd-00053 and Ssd-000106. CON III and PEP III samples were clustered into two groups, with the major group being closely associated with OTUs SSd-00048, OTUs SSd-00061 and OTUs SSd-00140, while the minor group was closely associated with OTU Ssd-00001. PEP II samples showed a very distinct distribution pattern, as half of the samples clustered with the CON II group, while the remaining samples were associated with the CON III - PEP III major cluster.

## Discussion

Products manufactured by hydrolysis of conventional protein ingredients have the potential to include bioactive peptides that can provide other functions or benefits in addition to supplying dietary amino acids. In this study, a commercial peptide-based additive, Peptiva, was tested as a possible source of bioactive molecules using two methods. First, its ability to compensate for reduced inclusion of dietary amino acid levels in weaned pig diets, by increasing the digestibility or the efficiency of use of protein ingredients, was assessed. In the context of a commercial swine production system as used in this study, there was no difference in performance during Phases I and II post-weaning, but PEP 10-fed pigs were found to weigh significantly less than CON-fed pigs by the end of Phase III. These results would indicate that, at least in the first 6 weeks post-weaning, Peptiva supplementation at low inclusion levels was not sufficient to compensate for a 10% reduction in dietary amino acid levels. Intriguingly, the average daily feed intake for pigs fed the PEP diet was lower than for pigs fed the CON diet during Phase III, but no differences in average daily gain were observed. While future research will be required to elucidate the possible mechanisms involved, perhaps the Peptiva formulation includes bioactive peptides that can improve the efficiency of nutrient utilization by the host, acting on the absorptive capacity of enterocytes or on their metabolism. Alternatively, Peptiva could act indirectly by modulating the activity of microbial symbionts. In addition, it is worth noting that 3% fewer pigs were removed from the trial, either because of mortality or illness, in the PEP-10 group compared to the other treatments, suggesting a benefit to overall pig health. Even if this observation is not supported by statistical testing, a lower removal rate in the absence of herd-wide antibiotic administration, as was implemented in this study, represents an important finding for the swine industry, since mass administration of antibiotics in the first 7 to 10 days following weaning is not allowed in conventional production.

The second potential activity of the Peptiva product investigated in this study was the ability to change or modulate the composition of the gut microbiome in weaned pigs. Since the composition of gut microbial communities has been associated with the health status and performance of individual hosts [3, 35–37], compounds that can change gut symbiont profiles have the potential to be developed as tools to improve critical livestock production parameters [38]. To this end, fecal bacterial communities were used as a proxy for gut microbiome composition analysis in weaned pigs, from which two main observations were made: evidence of bacterial succession between Phase II and Phase III in control-fed animals, and a stage-specific effect in Pep-fed pigs.

### Bacterial succession from Lactobacillaceae in phase II to Streptococcaceae in phase III

A comparison of the samples collected from control-fed pigs between Phase II and Phase III diets was suggestive of microbial succession, as major changes in taxonomic profiles and OTU composition were observed. For instance, members of the *Lactobacillaceae* family were found to be more abundant at the end of Phase II compared to the end of Phase III, which included four main OTUs (Ssd-0002, Ssd-00019, Ssd-00025, and Ssd-00053). In young animals, *Lactobacilli* have been reported to prevent adhesion of pathogens to the gut mucosa, inhibit growth of pathogens through production of lactate, and / or stimulate colonization of beneficial bacteria [9, 39–41]. Because of these types of activities, *Lactobacillus* species are considered beneficial to the gastrointestinal tract of animals, and are typically included in probiotic formulations. For instance, a probiotic formulation containing *L. gasseri*, *L. reuteri*, *L. acidophilus* and *L. fermentum* was reported to result in fewer incidences of diarrhea in weaned pigs and to lower *E. coli* counts after a pathogen challenge [39], while weaned pigs supplemented with *L. reuteri* were found to have higher average daily gain, longer ileal villi, as well as increased expression of the tight junction protein zonula occludens − 1 [42]. *Lactobacilli* have also been reported to have antimicrobial activity, as observed with *L. reuteri* which can inhibit the growth and mucosal adherence of enterotoxigenic *E. coli* [43], and *L. gasseri* which is known to produce a bacteriocin [44]. In the current study, three of the four most abundant *Lactobacillus*-affiliated OTUs were found to be closely related to *L. reuteri* or *L. gasseri.*

At the end of Phase III, members of the *Streptococcaceae* family became the most predominant bacterial group of the fecal microbiome in weaned pigs, while the abundance of *Lactobacillus*-affiliated bacteria was greatly reduced. Since the sequence identity to their respective closest *Streptococcus* relatives ranged between 90 and 96%, main OTUs Ssd-00039, Ssd-00048, Ssd-00061 and Ssd-00140 most likely corresponded to uncharacterized species of this genus*.* While the biological activities of *Streptococci* in the gut have not been as extensively studied as for *Lactobacilli,* members of this genus are also known to be lactate producers and to express bacteriocin, and thus could be involved in protection against pathogen proliferation in weaned pigs [45].

Of the factors that may be responsible for these observed changes in bacterial composition in pigs fed control diets, differences in diet formulation between Phase II and Phase III offer a reasonable explanation. Notably, three ingredients (dried whey, fish meal and zinc oxide) were included in Phase II diets, but not in Phase III diets (Supplementary File 1). As its primary use is to prevent diarrhea, zinc oxide represents a likely candidate modulator of gut microbiome composition [46–50]. However, its target bacterial groups in gut environments remain to be further investigated, as exemplified by two conflicting studies, one observing a decrease in *Lactobacilli* as a result of dietary inclusion of zinc oxide [48], while the other reported no effect [51]. Similarly, further investigations will be required to determine the effects of dried whey and fish meal, both used as high-quality protein ingredients, on the gut microbiome of weaned pigs.

### Stage-specific effect of Peptiva on the microbiome of weaned pigs

The second main observation from the comparative analysis of fecal bacterial communities performed in this study was that the profiles of PEP II samples appeared to be intermediate between CON II and CON III profiles. This was well illustrated by correspondence analysis, where PEP II samples appeared to be divided into two groups, with certain samples more similar to CON II profiles while others were more similar to CON III profiles. However, the analysis of additional samples would have been beneficial in providing increased resolution and confidence in support of this observation. At the OTU level, the respective abundances of Ssd-00019 and Ssd-00053 in PEP II were found to be statistically different from CON II (*P* < 0.05), but not from CON III. In contrast, no difference in abundance was found for Ssd-000140 between PEP II and CON II samples, which were however both significantly lower than those observed in the CON III samples. Other OTUs, such as Ssd-00002, Ssd-00025, Ssd-00039, Ssd-00048, Ssd-00061, and Ssd-00106, were found to be statistically different between CON II and CON III, while no significant pair-wise difference was found between either CON II and PEP II or between CON III and PEP II. Finally, no major differences in fecal bacterial profiles were observed between CON III and PEP III samples, indicating that both sets of fecal bacterial communities had reached similar compositional profiles. While additional research will be required to further elucidate the mechanisms responsible for these effects, the results presented in this study would suggest that Peptiva can promote maturation of swine fecal bacterial communities during a specific period of the nursery phase.

## Conclusions

Under the conditions tested in this study, Peptiva supplementation did not result in improved weight gains or feed efficiency of weaned pigs, but it was found to reduce average daily feed intake during Phase III of the nursery trial. In addition, the results presented in this report suggest that Peptiva can affect the fecal microbiome composition of animals during the first few weeks after weaning. In the context of the current understanding of gut microbiome development, early events that impact bacterial composition can have long term effects that persist in adults. For food animal production, this would suggest that development of practices or diet formulations that can establish more resistant, resilient and efficient gut microbiomes in neonates would provide lasting benefits into the growing and finishing stages. Based on their potential for producing a vast array of bioactive molecules, peptide formulations represent a largely untapped source of compounds that could be further developed into feed additives to benefit animal health and nutrition.

## Methods

### Animal performance trial and sample collection

The animal trial was conducted at the South Dakota State University (SDSU) Off-Site Wean-to-Finish Barn, with all procedures approved by the SDSU Institutional Animal Care and Use Committee before the start of the study (Protocol 17-035A). This swine facility is managed as a commercial-scale livestock barn to conduct nutritional and animal health research that can benefit producers in this sector. Weaned pigs (21 d of age, 5.6 ± 1.2 kg) were randomly allocated to 48 pens (24 pigs/pen), with each pen randomly assigned to one of three experimental diets (*n* = 16 pens/treatment): control diet (CON; formulated to meet the NRC (2012) nutrient requirements), Peptiva (PEP; control diet supplemented with Peptiva), and PEP with reduced amino acid content (PEP10; dietary amino acid content at 90% of NRC (2012) recommendations). All other dietary nutrients met or exceeded NRC (2012) recommendations for weaned pigs. Experimental diets were fed according to a standard nursery phase feeding program (Supplementary File 1): Phase I (d0-d7), Phase II (d8-d21), and Phase III (d22–42). Peptiva is a commercial product manufactured by Vitech Bio-Chem Corporation (Glendale, CA, USA) which consists of fish peptides, porcine digests and microbial peptides. In both PEP and PEP10 diets, Peptiva was included at 1, 0.5, and 0.3%, during Phases I, II, and III, respectively. The swine facility was divided into four blocks based on pen location within the barn, and each treatment was equally represented in each block (*n* = 4 pens/treatment/block). Pens of pigs were assigned to treatment within each block with consideration of pen weight to be equivalent between treatments as best as possible.

During the trial, one pen for the PEP group and one pen for the PEP10 group were fed the wrong diet. Consequently, these two pens were removed from the analysis, which was conducted using a total of 46 pens instead of 48 pens as originally planned (CON: *n* = 16; PEP: *n* = 15; PEP10: n = 15).

No mass antibiotic treatment via feed or water medicator was used during the course of the trial. Injectable antibiotics were only administered on an individual pig basis for treatment of scours or poor health. Individual pigs treated with injectable antibiotics that recovered from their symptoms remained in the performance trial, but they were not used to collect samples for composition analysis of fecal bacterial communities.

Body weights of the animals were measured by pen at the start of the trial, then on a weekly basis until the end of Phase III. Individual pig weights were determined at the beginning of the trial, at the end of Phase II and at the end of Phase III. Samples for microbiome analysis were collected at the end of Phase II and at the end of Phase III from ten animals fed the CON diets and ten individuals fed the PEP diet. More specifically, two representative individuals from each of five representative pens were selected for fecal sample collection for each diet. Pen weight was used to identify representative pens for each dietary treatment, and individual weight was used to identify representative animals from each selected pen. Fecal samples were collected by rectal palpation, then stored frozen (− 20 °C) until microbial genomic DNA extraction was performed.

At the conclusion of the trial, pens were randomly allotted to a separate grow finish trial, and the animals were marketed after achieving 130 kg body weight.

### Microbial DNA isolation and PCR amplification of the 16S rRNA gene

Microbial genomic DNA was isolated from fecal samples using the repeated bead beating plus column method, as previously described [52]. The V1-V3 region of the bacterial 16S rRNA gene was PCR-amplified using the 27F forward [53] and 519R reverse [54] primer pair. PCR reactions were performed with the Phusion Taq DNA polymerase (Thermo Scientific, Waltham, MA, USA) under the following conditions: hot start (4 min, 98 °C), followed by 35 cycles of denaturation (10 s, 98 °C), annealing (30 s, 50 °C) and extension (30 s, 72 °C), then ending with a final extension period (10 min, 72 °C). PCR products were separated by agarose gel electrophoresis, and amplicons of the expected size (~ 500 bp) were excised for gel purification using the QiaexII Gel extraction kit (Qiagen, Hilden, Germany). For each sample, approximately 400 ng of amplified DNA were submitted to Molecular Research DNA (MRDNA, Shallowater, TX, USA) for sequencing with the Illumina MiSeq (2X300) platform to generate overlapping paired end reads.

### Computational analysis of PCR generated 16S rRNA amplicon sequences

Unless specified, sequence data analysis was performed using custom written Perl scripts (Supplementary File 4). Raw bacterial 16S rRNA gene V1-V3 amplicon sequences were provided by Molecular Research DNA as assembled contigs from overlapping MiSeq (2X300) paired-end reads from the same flow cell clusters. Reads were then selected to meet the following criteria: presence of both intact 27F (forward) and 519R (reverse) primer nucleotide sequences, length between 400 and 580 nt, and a minimal quality threshold of no more than 1% of nucleotides with a Phred quality score lower than 15.

Following quality screens, sequence reads were aligned, then clustered into Operational Taxonomic Units (OTUs) at a genetic distance cutoff of 5% sequence dissimilarity [55]. While 3% is the most commonly used clustering cutoff for 16S rRNA, it was originally recommended for full length sequences, and may not be suitable for the analysis of specific subregions since nucleotide sequence variability is not constant across the entire length of the 16S rRNA gene. In this context, if 3% is a commonly accepted clustering cutoff for V4 or V4–V5 regions, which are the least variable of the hypervariable regions, then a higher cutoff should be used for the V1-V3 region, since V1 is the most variable region of the 16S rRNA gene. OTUs were screened for DNA sequence artifacts using the following methods. Chimeric sequences were first identified with the chimera.uchime and chimera.slayer commands from the MOTHUR open source software package [56]. Secondly, the integrity of the 5′ and 3′ ends of OTUs was evaluated using a database alignment search-based approach; when compared to their closest match of equal or longer sequence length from the NCBI nt database, as determined by BLAST [57], OTUs with more than five nucleotides missing from the 5′ or 3′ end of their respective alignments were discarded as artifacts. Single read OTUs were subjected to an additional screen, where only sequences that had a perfect or near perfect match to a sequence in the NCBI nt database were kept for analysis, i.e. that the alignment had to span the entire sequence of the OTU, and a maximum of 1% of dissimilar nucleotides was tolerated.

While a 5% cutoff may affect comparisons with other studies, its impact should be minimal. It would not be expected to affect the most abundant OTUs, which were the main focus of this study. While it would affect the total number of OTUs generated, the impact would be less than that of other practices that are commonly used in microbiome data analysis. For instance, the removal of all OTUs with a low number of reads would have had a greater impact, as these OTUs are by far the most abundant.

After removal of sequence chimeras and artifacts, taxonomic assignment of valid OTUs was determined using a combination of RDP Classifier [58] and BLAST [57]. The List of Prokaryotic Names with Standing in Nomenclature (LPSN - http://www.bacterio.net) was also consulted for information on valid species belonging to taxa of interest [59].

### Computational analysis for alpha and beta diversity

Using custom Perl scripts, all datasets were randomly rarefied to 3000 reads, which were then used to create ‘shared’-type formatted files. Ace, Chao1, Shannon and Simpson indices, as well as observed OTUs and coverage, were determined from the shared files using summary.single in MOTHUR [56]. For Principal Coordinate Analysis (PCoA), Bray-Curtis distances were first determined using summary.shared, which were then used as input for the command pcoa, with both procedure performed using MOTHUR [56]. Principal Components 1 (PC1) and 2 (PC2), representing the highest levels of variation, were plotted using Microsoft Excel. Correspondence Analysis (CA) was conducted in R (version 3.6.1) using the command ca from the R package ‘ca’. Outputs were plotted using Microsoft Excel.

### Statistical analyses

Analysis of performance data was performed using the PROC MIXED procedure of SAS (Version 9.4; SAS Inst. Inc., Cary, NC) with pen as the experimental unit and pen nested within block as the random variable. Dietary treatment was considered the fixed effect, and the effect of block was removed from the model because it was not significant. Data were a priori tested for normal distribution and homogeneity of variances. Initial body weight was used as covariate for analysis of weekly body weight. All possible comparisons between treatment means were tested using the PDiff option in SAS in combination with Tukey’s adjusted means separation when a significant main effect was observed, and data are presented as lsmeans +/− standard error of the mean. A Chi-squared test was used to evaluate the distribution of total pigs removed by treatment.

For comparison of bacterial taxonomic groups, alpha diversity indices, and OTU abundance amongst different sample groups (CON-Phase II, PEP-Phase II, CON-Phase III and PEP-Phase III), the non-parametric Friedman test (command friedman.test) and the post hoc Nemenyi test for multiple pairwise comparisons (command posthoc.friedman.nemenyi.test) were performed in R (Version R-3.2.3). Groups were considered to be significantly different when *P* ≤ 0.05.

### Accession numbers for next generation sequencing data

Raw sequence data are available from the NCBI Sequence Read Archive under Bioproject PRJNA533644 and SRA accession SRP192997.

## Supplementary information

**Additional file 1: Supplementary File 1.** Ingredient composition of experimental diets. List of ingredients and their respective proportion (%) in each nursery pig phase diet used in this study.

**Additional file 2: Supplementary File 2.**. Operational Taxonomic Unit (OTU) table. Relative abundance for each OTU identified in fecal samples collected from nursery pigs fed a control (CON) or Peptiva-supplemented diet (PEP) at two different stages.

**Additional file 3: Supplementary File 3.***P* values associated with Table 3 and Table 5. Tables showing the *P* values obtained from the non-parametric Friedman test and the post hoc Nemenyi test for multiple pairwise comparisons that are presented in Table 3 and Table 5.

**Additional file 4: Supplementary File 4.** Custom Perl script pipeline for 16S rRNA data analysis. List of Perl scripts and their respective code that were used for analysis of 16S rRNA data.

## Data Availability

Raw sequence data are available from the NCBI Sequence Read Archive under Bioproject PRJNA533644 and SRA accession SRP192997.

## Abbreviations

16S rRNA: 16S ribosomal RNA
BLAST: Basic Local Alignment Search Tool
CON II: Samples collected at the end of Phase II from animals fed the control diet
CON III: Samples collected at the end of Phase III from animals fed the control diet
CON: Control diet
LPSN: List of Prokaryotic Names with Standing in Nomenclature
NCBI: National Center for Biotechnology Information
OTU: Operational Taxonomic Unit
PCoA: Principal Coordinate Analysis
PEP II: Samples collected at the end of Phase II from animals fed the Peptiva diet
PEP III: Samples collected at the end of Phase III from animals fed the Peptiva diet
PEP: Control diet supplemented with Peptiva
PEP10: Control diet containing 90% of the recommended amino acid levels, supplemented with Peptiva
RDP: Ribosomal Database Project
SDSU: South Dakota State University
