# Supplementary material for: Dietary inclusion of Peptiva, a peptide-based feed additive, can accelerate the maturation of the fecal bacterial microbiome in weaned pigs
Source: BMC Vet Res. 2020 Feb 18;16:60. doi: 10.1186/s12917-020-02282-x (PMC7026967; doi:10.1186/s12917-020-02282-x)
Supplement: Supplementary file 1 — Additional file 1: Supplementary File 1. Ingredient composition of experimental diets. List of ingredients and their respective proportion (%) in each nursery pig phase diet used in this study. [file 12917_2020_2282_MOESM1_ESM.pdf]

**Supplementary File 1. Ingredient composition of experimental diets.**

List of ingredients and their respective proportion (%) in each nursery pig phase diet used in this study.

| | Phase 1 | | | Phase 2 | | | Phase 3 | | |
|---|---|---|---|---|---|---|---|---|---|
| **Item** | Control | Peptiva | Peptiva-10 | Control | Peptiva | Peptiva-10 | Control | Peptiva | Peptiva-10 |
| **Corn** | 35.8 | 35.7 | 41.7 | 54.2 | 54.2 | 59.4 | 66.0 | 66.0 | 71.0 |
| **SBM, 46.5%** | 19.5 | 18.5 | 12.5 | 27.7 | 27.2 | 22.0 | 30.4 | 30.1 | 25.0 |
| **Dried whey** | 25 | 25 | 25 | 10 | 10 | 10 | 0 | 0 | 0 |
| **Menhaden FM** | 7 | 7 | 7 | 3 | 3 | 3 | 0 | 0 | 0 |
| **HP-300** | 7 | 7 | 7 | 0 | 0 | 0 | 0 | 0 | 0 |
| **L-Lys** | 0.26 | 0.26 | 0.25 | 0.32 | 0.32 | 0.32 | 0.4 | 0.4 | 0.4 |
| **DL-Met** | 0.2 | 0.2 | 0.17 | 0.15 | 0.15 | 0.12 | 0.15 | 0.15 | 0.13 |
| **L-Thr** | 0.11 | 0.11 | 0.1 | 0.12 | 0.12 | 0.11 | 0.15 | 0.15 | 0.14 |
| **L-Trp** | 0.03 | 0.03 | 0.03 | 0.03 | 0.03 | 0.04 | 0 | 0 | 0.01 |
| **Soybean oil** | 3.7 | 3.7 | 3.7 | 2 | 2 | 2 | 0 | 0 | 0 |
| **Mono cal** | 0.5 | 0.45 | 0.5 | 1.1 | 1.08 | 1.12 | 1.41 | 1.41 | 1.41 |
| **Limestone** | 0.32 | 0.33 | 0.35 | 0.85 | 0.85 | 0.87 | 1.02 | 1.02 | 1.05 |
| **Salt** | 0.03 | 0.03 | 0.03 | 0.03 | 0.03 | 0.03 | 0.35 | 0.35 | 0.35 |
| **Zinc oxide, 72%** | 0.42 | 0.42 | 0.42 | 0.28 | 0.28 | 0.28 | 0 | 0 | 0 |
| **Vit pmx** | 0.05 | 0.05 | 0.05 | 0.05 | 0.05 | 0.05 | 0.05 | 0.05 | 0.05 |
| **Min pmx** | 0.15 | 0.15 | 0.15 | 0.15 | 0.15 | 0.15 | 0.15 | 0.15 | 0.15 |
| **Peptiva** | 0 | 1.0 | 1.0 | 0 | 0.5 | 0.5 | 0 | 0.3 | 0.3 |
