# Supplementary material for: Dietary inclusion of Peptiva, a peptide-based feed additive, can accelerate the maturation of the fecal bacterial microbiome in weaned pigs
Source: BMC Vet Res. 2020 Feb 18;16:60. doi: 10.1186/s12917-020-02282-x (PMC7026967; doi:10.1186/s12917-020-02282-x)
Supplement: Supplementary file 4 — Additional file 4: Supplementary File 4. Custom Perl script pipeline for 16S rRNA data analysis. List of Perl scripts and their respective code that were used for analysis of 16S rRNA data. [file 12917_2020_2282_MOESM4_ESM.pdf]

Supplementary File 4. Custom Perl script pipeline for 16S rRNA data analysis.

List of the Perl scripts in the order in which they were used. The basic procedure consists of using the output of a script as the input for the next script.

Quality filtering
MiSeq FOR-REV primer screen and trim at REV primer.pl
MiSeq length selection.pl
MiSeq qual screening based on quality threshold.pl
MiSeq fasta retrieving reads from accnos file.pl
MiSeq read name change based on barcode.pl
Fasta to phylip conversion.pl
MiSeq removing barcodes from sequences.pl

Alignment of 16S rRNA sequences (V1-V3)
16S C1 alignment.pl
16S C1 alignment check.pl
16S C2 alignment.pl
16S C2 alignment check.pl
16S C3 begin alignment.pl
16S C3 begin alignment check.pl
16S C3 end alignment.pl
16S C4 alignment.pl
16S C4 trim end sequence trim.pl
16S alignment length adjustment.pl

Clustering
OTU clustering_phylip format.pl

```
#####################################  MiSeq FOR-REV primer screen and trim at REV primer.pl

$INPUT_FASTA_FILE = "NAME OF INPUT FILE" ;

@ARRAY_INPUT_FILE = split('\.',$INPUT_FASTA_FILE) ;
$NAME_OF_FILE = $ARRAY_INPUT_FILE[0] ;

$BARCODE_FORWARD_PRIMER = "AG.GTTTGATC.TGGCTCAG" ; #27F

$REVERSE_PRIMER = "CAGC.GCCGC.GTAA" ; #519R
$LENGTH_TO_SEARCH = 100 ;
$OUTPUT = ""  ;

print "Screening for forward and reverse primer sequences\n" ;
$COUNT = 0 ;
open (FASTA, "$INPUT_FASTA_FILE") or die "Can't open $file for reading" ;
while (<FASTA>)        {
       my $LINE = $_; chomp ($LINE);
       if ($LINE =~ />/)      {
              $COUNT = $COUNT + 1 ;  my $COUNT_THOUSAND = $COUNT/20000 ;
              my $COUNT_INTEGER = int($COUNT_THOUSAND) ; my $INTEGER_CHECK = $COUNT_THOUSAND -
$COUNT_INTEGER;
              if ($INTEGER_CHECK == 0) {print "Screening sequence $COUNT\n";      }

              my $NAME = $LINE ;
              my $SEQUENCE = <FASTA> ; chomp ($SEQUENCE) ;
              my $LENGTH_SEQ = length($SEQUENCE) ;

              my $UPSTREAM_REGION = substr($SEQUENCE,0,$LENGTH_TO_SEARCH);
              my $UPSTREAM_FOUND = 0 ;
              if ($UPSTREAM_REGION =~ /$BARCODE_FORWARD_PRIMER/) {$UPSTREAM_FOUND = 2 ;}

              my $DOWNSTREAM_START = $LENGTH_SEQ - $LENGTH_TO_SEARCH ;
              my $DOWNSTREAM_REGION = substr($SEQUENCE,$DOWNSTREAM_START,$LENGTH_TO_SEARCH);
              my $DOWNSTREAM_FOUND = 0 ;
              if ($DOWNSTREAM_REGION =~ /$REVERSE_PRIMER/) {$DOWNSTREAM_FOUND = 2 ;}

              if ($UPSTREAM_FOUND > 1 and $DOWNSTREAM_FOUND > 1)   {
#trimming sequence at rev primer
                     my $THREE_PRIME_FOUND = 0 ; my $THREE_PRIME_BOUNDARY = 0 ;
                     my $LENGTH_TO_SEARCH = length($REVERSE_PRIMER) ;
                     my $DOWNSTREAM_SCREEN_START = $LENGTH_SEQ - $LENGTH_TO_SEARCH ;
                     while ($THREE_PRIME_FOUND < 1)        {
                            my $DOWNSTREAM_SEARCH = substr($SEQUENCE, $DOWNSTREAM_SCREEN_START,
$LENGTH_TO_SEARCH) ;
                            if ($DOWNSTREAM_SEARCH !~ /$REVERSE_PRIMER/) {
                                   $DOWNSTREAM_SCREEN_START = $DOWNSTREAM_SCREEN_START - 1 ;

                     }
                            if ($DOWNSTREAM_SEARCH =~ /$REVERSE_PRIMER/) {
                                   $THREE_PRIME_FOUND =  2 ;
                                   $THREE_PRIME_BOUNDARY = $DOWNSTREAM_SCREEN_START +
$LENGTH_TO_SEARCH ;

                     }

                                                                               }
                     my $TRIMMED_SEQUENCE = substr($SEQUENCE,0,$THREE_PRIME_BOUNDARY) ;
                     $OUTPUT = $OUTPUT.="$NAME\n$TRIMMED_SEQUENCE\n" ;

              }
                                          }
                                   }
close (FASTA) ;


open (OUTPUT, ">OUTPUT_$NAME_OF_FILE\_FOR_REV_trim.fasta") or die "Can't open $file for reading"
;
print OUTPUT "$OUTPUT" ;
close (OUTPUT);
```

```
#####################################  MiSeq length selection.pl

$INPUT_FILE_NAME = "NAME OF INPUT FILE" ;

@ARRAY_INPUT_FILE = split('\.',$INPUT_FILE_NAME) ;
$NAME_OF_FILE = $ARRAY_INPUT_FILE[0] ;

print"Setting up hash\n" ;
%HASH_LENGTH ;
$MAX_LENGTH = 600 ;
$MIN_LENGTH = 400;

open (FASTA, "$INPUT_FILE_NAME") or die "Can't open $file for reading" ;
$READ_COUNT = 0 ; $LONGEST_READ = 0 ; $OUTPUT = "" ;
while (<FASTA>) {
	$READ_COUNT = $READ_COUNT + 1 ; my $COUNT_THOUSAND = $READ_COUNT/2000 ;
	my $COUNT_INTEGER = int($COUNT_THOUSAND) ; my $INTEGER_CHECK = $COUNT_THOUSAND -
$COUNT_INTEGER;
	if ($INTEGER_CHECK == 0) {print "Processing sequence $READ_COUNT\n";	}

	my $LINE = $_ ; chomp ($LINE) ;
	if ($LINE =~ />/)	{
		my $READ_NAME = $LINE ;
		my $SEQ = <FASTA>;  chomp($SEQ) ;
		my $LENGTH_SEQ = length($SEQ) ;

		if ($LENGTH_SEQ > $MIN_LENGTH and $LENGTH_SEQ < $MAX_LENGTH)	{
			$OUTPUT  = $OUTPUT.="$READ_NAME\n$SEQ\n" ;

	}
					}
				}
close (FASTA) ;

print "Preparing output\n" ;

open (OUTPUT, ">OUTPUT_$NAME_OF_FILE\_$MIN_LENGTH-$MAX_LENGTH\_nt.fasta") or die "Can't open
$file for reading" ;
print OUTPUT "$OUTPUT" ;
close (OUTPUT);
```

```
######################################  MiSeq qual screening based on quality threshold.pl
$INPUT_FASTA_FILE = " NAME OF INPUT FILE " ;
$INPUT_QUAL_FILE = " NAME OF INPUT FILE" ;

@ARRAY_INPUT_FASTA_NAME = split('\.',$INPUT_FASTA_FILE) ;
$INPUT_FASTA_NAME_NO_EXTENSION = $ARRAY_INPUT_FASTA_NAME[0] ;

$QUALITY_THRESHOLD = 15 ; $MAX_NUMBER_NT_BELOW_THRESHOLD = 5 ;
$LOW_QUAL_NT_COUNT_MAX = 0 ;
%HASH_QUAL ; %HASH_SURVEY_QUAL ;
$OUTPUT = "" ; $OUTPUT_SUMMARY = "" ;
$OUTPUT_ERROR = "Inconsistency between fasta and qual for reads:\n" ;

open (QUAL, "$INPUT_QUAL_FILE") or die "Can't open $file for reading" ;
$READ_COUNT = 0 ;
while (<QUAL>)        {
       my $LINE = $_ ;       chomp ($LINE);
       if ($LINE =~ />/)             {
              $READ_COUNT = $READ_COUNT + 1 ; my $COUNT_THOUSAND = $READ_COUNT/20000 ;
              my $COUNT_INTEGER = int($COUNT_THOUSAND) ; my $INTEGER_CHECK = $COUNT_THOUSAND -
$COUNT_INTEGER;
              if ($INTEGER_CHECK == 0) {print "Loading quality for $READ_COUNT\n";       }

              my @ARRAY_LINE = split(' ',$LINE) ;
              my $NAME = $ARRAY_LINE[0] ;
              my $QUAL_DATA_LINE = <QUAL> ; chomp ($QUAL_DATA_LINE) ;
              $HASH_QUAL{$NAME} = $QUAL_DATA_LINE ;
                                                 }
                            }
close (QUAL);

open (FASTA, "$INPUT_FASTA_FILE") or die "Can't open $file for reading" ;
$READ_COUNT_1 = 0 ;
print "Screening fasta for quality\n" ;
while (<FASTA>)       {
       my $LINE = $_ ;       chomp ($LINE);
       if ($LINE =~ />/)      {
              $READ_COUNT_1 = $READ_COUNT_1 + 1 ; my $COUNT_THOUSAND = $READ_COUNT_1/20000 ;
              my $COUNT_INTEGER = int($COUNT_THOUSAND) ; my $INTEGER_CHECK = $COUNT_THOUSAND -
$COUNT_INTEGER;
              if ($INTEGER_CHECK == 0) {print "Checking read $READ_COUNT_1 for quality\n";
       }

              my @ARRAY_LINE = split(' ',$LINE) ;
              my $NAME = $ARRAY_LINE[0] ;
              my $SEQ = <FASTA> ; chomp($SEQ) ;
              my $LENGTH_SEQ = length($SEQ) ;

              my $QUALITY_DATA = $HASH_QUAL{$NAME} ;
              my @ARRAY_QUALITY_DATA = split(' ',$QUALITY_DATA);
              my $NUMBER_QUAL_DATA_ENTRIES = scalar(@ARRAY_QUALITY_DATA);
              if ($NUMBER_QUAL_DATA_ENTRIES < $LENGTH_SEQ)        { $OUTPUT_ERROR =
$OUTPUT_ERROR.="$NAME," ; }

              my $QUAL_COUNT = 0 ; my $LOW_QUAL_NT_COUNT = 0 ;
              while ($QUAL_COUNT < $LENGTH_SEQ) {
                     my $QUAL_SCORE = $ARRAY_QUALITY_DATA[$QUAL_COUNT] ;
                     if ($QUAL_SCORE < $QUALITY_THRESHOLD)        {
                            $LOW_QUAL_NT_COUNT = $LOW_QUAL_NT_COUNT + 1 ;

       }
                     $QUAL_COUNT = $QUAL_COUNT + 1 ;

                                                                                    }

              if ($LOW_QUAL_NT_COUNT <= $MAX_NUMBER_NT_BELOW_THRESHOLD)    {
                     $OUTPUT = $OUTPUT.="$NAME\n" ;


                            }
              my $UPDATING_LOW_QUAL_SURVEY = $HASH_SURVEY_QUAL{$LOW_QUAL_NT_COUNT} ;
```

```
               my $UPDATING_LOW_QUAL_SURVEY = $UPDATING_LOW_QUAL_SURVEY.="$NAME," ;
               $HASH_SURVEY_QUAL{$LOW_QUAL_NT_COUNT} = $UPDATING_LOW_QUAL_SURVEY  ;

               if ($LOW_QUAL_NT_COUNT > $LOW_QUAL_NT_COUNT_MAX) {$LOW_QUAL_NT_COUNT_MAX =
$LOW_QUAL_NT_COUNT ; }
                                             }
                                      }
close (FASTA) ;
print "Preparing summary\n" ;
$OUTPUT_SUMMARY = $OUTPUT_SUMMARY."The highest number of lower threshold nt is
$LOW_QUAL_NT_COUNT_MAX\n" ;
$COUNT_LOW_QUAL_SURVEY = $LOW_QUAL_NT_COUNT_MAX ;
while ($COUNT_LOW_QUAL_SURVEY >= 0)   {
       my $INFO_TO_TRANSFER = $HASH_SURVEY_QUAL{$COUNT_LOW_QUAL_SURVEY} ;
       my @ARRAY_INFO_TO_TRANSFER = split(',',$INFO_TO_TRANSFER ) ;
       my $NUMBER_NAMES = scalar(@ARRAY_INFO_TO_TRANSFER) ;
       if ($NUMBER_NAMES > 0)        {
               $OUTPUT_SUMMARY = $OUTPUT_SUMMARY."Reads with $COUNT_LOW_QUAL_SURVEY nt below
threshold:\t$NUMBER_NAMES\n" ;
                                                            }
       $COUNT_LOW_QUAL_SURVEY = $COUNT_LOW_QUAL_SURVEY - 1 ;
                                                                           }

open (OUTPUT, ">OUTPUT_$INPUT_FASTA_NAME_NO_EXTENSION\_Q$QUALITY_THRESHOLD\_reads\.accnos") or
die "Can't open $file for reading" ;
print OUTPUT "$OUTPUT_ERROR\n\n" ;
print OUTPUT "$OUTPUT_SUMMARY\n\n" ;
print OUTPUT "$OUTPUT" ;
close (OUTPUT);
```

```
###################################### MiSeq fasta retrieving reads from accnos file.pl
$INPUT_FILE_FASTA = " NAME OF INPUT FILE " ;

$INPUT_FILE_ACCNOS = " NAME OF INPUT FILE " ;
@ARRAY_ACCNOS = split('\.',$INPUT_FILE_ACCNOS) ;
$NAME_ACCNOS_NO_EXTENSION = $ARRAY_ACCNOS[0] ;

$P = "P" ; $p = "p" ; $F = "F" ; $f = "f" ;
print "Would you like a phylip (P) or fasta (F) output?\n" ;
$FASTA_OR_PHYLIP = <> ;
$INPUT_FORMAT_CHECK = 0 ;
while ($INPUT_FORMAT_CHECK < 1) {
        if ($FASTA_OR_PHYLIP =~ /P|p|F|f/)    {
                $INPUT_FORMAT_CHECK = 2 ;
                                                                                }
        if ($FASTA_OR_PHYLIP !~ /P|p|F|f/)    {
                print "Please enter P (phylip) or F (fasta)\n" ;
                $FASTA_OR_PHYLIP = <> ;
                                                                                }
                                                                }

open (FASTA, "$INPUT_FILE_FASTA") or die "Can't open $file for reading" ;
print "Loading fasta data in hash\n" ;
$READ_COUNT = 0 ; %FASTA_HASH ;
while (<FASTA>) {
        my $FASTA_LINE = $_ ; chomp ($FASTA_LINE) ;
        if ($FASTA_LINE =~ />/) {
                $READ_COUNT = $READ_COUNT + 1 ; my $COUNT_THOUSAND = $READ_COUNT/100000 ;
                my $COUNT_INTEGER = int($COUNT_THOUSAND) ; my $INTEGER_CHECK = $COUNT_THOUSAND -
$COUNT_INTEGER;
                if ($INTEGER_CHECK == 0) {print "Processing sequence $READ_COUNT\n";       }

                my @ARRAY_LINE = split(' ',$FASTA_LINE) ;
                my $FASTA_NAME = $ARRAY_LINE[0] ;

                my $FASTA_SEQ = <FASTA> ; chomp ($FASTA_SEQ) ;
                $FASTA_HASH{$FASTA_NAME} = $FASTA_SEQ ;
                                                        }
                                }
close (FASTA) ;
print "Finished loading fasta data in hash\n" ;

open (LIST, "$INPUT_FILE_ACCNOS") or die "Can't open $file for reading" ;
$OUTPUT = "" ;          $LINE_COUNT = 0 ;

while (<LIST>) {
        $LINE_COUNT = $LINE_COUNT + 1 ; my $COUNT_THOUSAND = $LINE_COUNT/100000 ;
        my $COUNT_INTEGER = int($COUNT_THOUSAND) ; my $INTEGER_CHECK = $COUNT_THOUSAND -
$COUNT_INTEGER;
        if ($INTEGER_CHECK == 0) { print "Processing line $LINE_COUNT\n" ; }

        my $LINE = $_ ; chomp($LINE) ;
        my $QUERY_NAME = $LINE ;
        if ($QUERY_NAME !~ /Reads with/) {
                if ($QUERY_NAME !~ />/) {
                        $QUERY_NAME = ">$QUERY_NAME" ;
                                                                }
                my $SEQUENCE = $FASTA_HASH{$QUERY_NAME} ;
                if ($FASTA_OR_PHYLIP =~ /$F/ or $FASTA_OR_PHYLIP =~ /$f/) {
                        $OUTPUT = $OUTPUT.="$QUERY_NAME\n$SEQUENCE\n" ;

                        }
                if ($FASTA_OR_PHYLIP =~ /$P/ or $FASTA_OR_PHYLIP =~ /$p/) {
                        $OUTPUT = $OUTPUT.="$QUERY_NAME\t$SEQUENCE\n" ;

                        }
                                                                                }
                                }
close (LIST) ;

if ($FASTA_OR_PHYLIP =~ /$F/ or $FASTA_OR_PHYLIP =~ /$f/) {
```

```
	open (OUTPUT, ">OUTPUT_retrieved_reads_$NAME_ACCNOS_NO_EXTENSION\.fasta") or die "Can't
open $file for reading" ;
	print OUTPUT "$OUTPUT" ;
	close (OUTPUT);

	}
if ($FASTA_OR_PHYLIP =~ /$P/ or $FASTA_OR_PHYLIP =~ /$p/) {
	open (OUTPUT, ">OUTPUT_retrieved_reads_$NAME_ACCNOS_NO_EXTENSION\.phylip") or die "Can't
open $file for reading" ;
	print OUTPUT "$OUTPUT" ;
	close (OUTPUT);

	}
```

```
###################################### MiSeq read name change based on barcode.pl

$INPUT_SAMPLEID_BARCODES = " NAME OF INPUT FILE " ;
#NAME OF FILE WITH SAMPLE IDs AND BARCODES AS SEPARATE COLUMNS
$INPUT_QUAL_CHECKED_FASTA = " NAME OF INPUT FILE " ;

print "Uploading sampleID and barcode information\n" ;
%BARCODE_HASH ; $BARCODE_LIST = "" ; $BARCODE_LENGTH = 0 ;  $BARCODE_START_POSITION = 0 ;

open (SAMPLE_ID_FILE, "$INPUT_SAMPLEID_BARCODES") or die "Can't open $file for reading" ;
 while (<SAMPLE_ID_FILE>)     {
        my $SAMPLE_ID_LINE = $_; chomp($SAMPLE_ID_LINE) ;
        if ($SAMPLE_ID_LINE !~ /SampleID/)    {
                my @ARRAY_SAMPLE_ID_LINE = split('\t', $SAMPLE_ID_LINE) ;
                my $SAMPLE_ID = $ARRAY_SAMPLE_ID_LINE[0] ; my $BARCODE = $ARRAY_SAMPLE_ID_LINE[1]
;
                $BARCODE_LENGTH = length($BARCODE) ; my $NUMBER_OF_READS = 0 ; my $RENAME_COUNT =
0 ;
                $BARCODE_LIST = $BARCODE_LIST.="$BARCODE\t" ;
                $BARCODE_HASH{$BARCODE} = "$SAMPLE_ID\t$NUMBER_OF_READS\t$RENAME_COUNT" ;
                                                                                }
                                        }
close (SAMPLE_ID_FILE) ;

print "Counting number of reads for each barcode\n" ; #COUNTING NUMBER OF READS FOR EACH BARCODE
$COUNT = 0 ;
open (FILE, "$INPUT_QUAL_CHECKED_FASTA") or die "Can't open $file for reading" ;
while (<FILE>)         {
        my $LINE = $_; chomp ($LINE);
        if ($LINE =~ />/)      {
                $COUNT = $COUNT + 1 ;  my $COUNT_THOUSAND = $COUNT/20000 ;
                my $COUNT_INTEGER = int($COUNT_THOUSAND) ; my $INTEGER_CHECK = $COUNT_THOUSAND -
$COUNT_INTEGER;
                if ($INTEGER_CHECK == 0) {print "Counting reads per barcode, sequence $COUNT\n";
        }

                my $SEQUENCE = <FILE> ; chomp ($SEQUENCE) ;
                my $LENGTH_SEQUENCE = length($SEQUENCE) ;
                if ($LENGTH_SEQUENCE > 10) {
                        my $SEQUENCE_BARCODE =
substr($SEQUENCE,$BARCODE_START_POSITION,$BARCODE_LENGTH);
                        my $RETRIEVED_HASH_LINE = $BARCODE_HASH{$SEQUENCE_BARCODE} ;
                        my @HASH_LINE_ARRAY = split('\t', $RETRIEVED_HASH_LINE) ;
                        my $SAMPLE_ID = $HASH_LINE_ARRAY[0] ; my $RENAME_COUNT = $HASH_LINE_ARRAY
[2] ;
                        my $NUMBER_OF_READS = $HASH_LINE_ARRAY [1] ;
                        $NUMBER_OF_READS = $NUMBER_OF_READS + 1;
                        $BARCODE_HASH{$SEQUENCE_BARCODE} =
"$SAMPLE_ID\t$NUMBER_OF_READS\t$RENAME_COUNT" ;
                                                                        }
                                                }
                                        }
close (FILE) ;

print "Renaming reads\n" ;
$RENAMED_READS = "" ; $NAME_MATCHING = "" ;
$COUNT = 0 ;
open (FILE_2, "$INPUT_QUAL_CHECKED_FASTA") or die "Can't open $file for reading" ;
while (<FILE_2>)               {
        my $LINE = $_; chomp ($LINE);
        if ($LINE =~ />/)      {
                $COUNT = $COUNT + 1 ;  my $COUNT_THOUSAND = $COUNT/20000 ;
                my $COUNT_INTEGER = int($COUNT_THOUSAND) ; my $INTEGER_CHECK = $COUNT_THOUSAND -
$COUNT_INTEGER;
                if ($INTEGER_CHECK == 0) {print "Renaming reads, sequence $COUNT\n";       }

                my $NAME = $LINE ; my $SEQUENCE = <FILE_2> ; chomp ($SEQUENCE) ;
                my $LENGTH_SEQUENCE = length($SEQUENCE) ;
                if ($LENGTH_SEQUENCE > 10) {
                        my $SEQUENCE_BARCODE =
substr($SEQUENCE,$BARCODE_START_POSITION,$BARCODE_LENGTH);
```

```
                      my $RETRIEVED_HASH_LINE = $BARCODE_HASH{$SEQUENCE_BARCODE} ;
                      my @HASH_LINE_ARRAY = split('\t', $RETRIEVED_HASH_LINE) ;
                      my $SAMPLE_ID = $HASH_LINE_ARRAY[0] ; my $NUMBER_OF_READS =
$HASH_LINE_ARRAY [1] ;
                      my $PADDING_NUMBER = 0 ;
                      if ($NUMBER_OF_READS < 100) { $PADDING_NUMBER = 2 ;}
                      if ($NUMBER_OF_READS >= 100 && $NUMBER_OF_READS < 1000) {$PADDING_NUMBER =
3 ; }
                      if ($NUMBER_OF_READS >= 1000 && $NUMBER_OF_READS < 10000) {$PADDING_NUMBER
= 4 ; }
                      if ($NUMBER_OF_READS >= 10000 && $NUMBER_OF_READS < 100000)
{$PADDING_NUMBER = 5 ; }
                      if ($NUMBER_OF_READS >= 100000 && $NUMBER_OF_READS < 1000000)
{$PADDING_NUMBER = 6 ; }
                      if ($NUMBER_OF_READS >= 1000000 && $NUMBER_OF_READS < 10000000)
{$PADDING_NUMBER = 7 ; }
                      if ($NUMBER_OF_READS >= 10000000 && $NUMBER_OF_READS < 100000000)
{$PADDING_NUMBER = 8 ; }

                      my $RENAME_COUNT = $HASH_LINE_ARRAY[2] ;
                      $RENAME_COUNT = $RENAME_COUNT + 1;
                      my $PADDED_COUNT = sprintf("%0$PADDING_NUMBER.f", $RENAME_COUNT) ;
                      my $NEW_NAME = ">$SAMPLE_ID-$PADDED_COUNT" ;

                      $RENAMED_READS = $RENAMED_READS.="$NEW_NAME\n$SEQUENCE\n";

                      $NAME_MATCHING = $NAME_MATCHING.="$NEW_NAME \t$NAME\n" ;

                      $BARCODE_HASH{$SEQUENCE_BARCODE} =
"$SAMPLE_ID\t$NUMBER_OF_READS\t$RENAME_COUNT" ;
                                                                            }
                                                  }
                                                  }
close (FILE_2);

open (OUTPUT, ">OUTPUT_renamed_$INPUT_QUAL_CHECKED_FASTA") or die "Can't open $file for reading"
;
print OUTPUT "$RENAMED_READS" ;
close (OUTPUT);

open (OUTPUT_NAME, ">OUTPUT_matching_original_names_$INPUT_QUAL_CHECKED_FASTA\.name") or die
"Can't open $file for reading" ;
print OUTPUT_NAME "$NAME_MATCHING" ;
close (OUTPUT_NAME);

my @BARCODE_LIST_ARRAY = split('\t', $BARCODE_LIST) ;
my $NUMBER_OF_BARCODES = scalar(@BARCODE_LIST_ARRAY) ;
$COUNT = 0 ; $OUTPUT_SUMMARY = "" ;
while ($COUNT < $NUMBER_OF_BARCODES)  {
       my $BARCODE = $BARCODE_LIST_ARRAY[$COUNT] ;
       my $RETRIEVED_HASH_LINE = $BARCODE_HASH{$BARCODE} ;
       my @HASH_LINE_ARRAY = split('\t', $RETRIEVED_HASH_LINE) ;
       my $SAMPLE_ID = $HASH_LINE_ARRAY[0] ;
       my $NUMBER_OF_READS = $HASH_LINE_ARRAY [1] ;
       $OUTPUT_SUMMARY = $OUTPUT_SUMMARY.="There are $NUMBER_OF_READS reads for sample
$SAMPLE_ID (barcode $BARCODE)\n" ;
       $COUNT = $COUNT + 1 ;
                                                                           }

open (OUTPUT_SUMMARY, ">OUTPUT_summary_of_renamed_reads_$INPUT_QUAL_CHECKED_FASTA\.txt") or die
"Can't open $file for reading" ;
print OUTPUT_SUMMARY "$OUTPUT_SUMMARY" ;
close (OUTPUT_SUMMARY);
```

```
#####################################       Fasta to phylip conversion.pl
$INPUT_FILE_NAME = " NAME OF INPUT FILE" ;
@ARRAY_FILE_NAME = split('\.',$INPUT_FILE_NAME) ;

$FILE_NAME_NO_EXTENSION = $ARRAY_FILE_NAME[0] ;



open (FASTA, "$INPUT_FILE_NAME") or die "Can't open $file for reading" ;



$READ_COUNT = 0 ; $OUTPUT = "" ;

while (<FASTA>)        {

        my $LINE = $_ ;        chomp ($LINE);

        if ($LINE =~ />/)      {

                $READ_COUNT = $READ_COUNT + 1 ; my $COUNT_THOUSAND = $READ_COUNT/10000 ;

                my $COUNT_INTEGER = int($COUNT_THOUSAND) ; my $INTEGER_CHECK = $COUNT_THOUSAND -
$COUNT_INTEGER;

                if ($INTEGER_CHECK == 0) {print "Checking sequence $READ_COUNT\n";  }


                if ($LINE =~ />/)      {

                        my $NAME = $LINE ;

                        my $SEQUENCE = <FASTA> ; chomp ($SEQUENCE) ;

                        $OUTPUT = $OUTPUT.="$NAME\t$SEQUENCE\n";

                                                        }

                                                }

                                        }

close (FASTA);

open (OUTPUT, ">OUTPUT_converted_to_phylip_$FILE_NAME_NO_EXTENSION.phylip") or die "Can't open
$file for reading" ;

print OUTPUT "$OUTPUT";

close (OUTPUT);
```

```
######################################      MiSeq removing barcodes from sequences.pl
$INPUT_FILE_NAME = " NAME OF INPUT FILE" ;
open (FILE, "$INPUT_FILE_NAME") or die "Can't open $file for reading" ;

@ARRAY_FILE_NAME = split('\.',$INPUT_FILE_NAME) ;
$FILE_NAME_NO_EXTENSION = $ARRAY_FILE_NAME[0] ;

$LENGTH_OF_BARCODE = 8 ;      #####################################

$READ_COUNT = 0 ;
$OUTPUT = "";

while (<FILE>)        {
        $READ_COUNT = $READ_COUNT + 1 ; my $COUNT_THOUSAND = $READ_COUNT/5000 ;
        my $COUNT_INTEGER = int($COUNT_THOUSAND) ; my $INTEGER_CHECK = $COUNT_THOUSAND -
$COUNT_INTEGER;
        if ($INTEGER_CHECK == 0) {print "Checking sequence $READ_COUNT\n";  }

        my $LINE = $_;        chomp ($LINE);

        my @ARRAY_LINE = split('\t', $LINE) ;
        my $NAME = $ARRAY_LINE[0] ; my $SEQ = $ARRAY_LINE[1] ;
        my $SEQ_LENGTH = length($SEQ) ;
        my $SEQ_LENGTH_NO_BARCODE = $SEQ_LENGTH - $LENGTH_OF_BARCODE ;
        my $NO_BARCODE_SEQ = substr($SEQ, $LENGTH_OF_BARCODE, $SEQ_LENGTH_NO_BARCODE);
        $OUTPUT = $OUTPUT.="$NAME\t$NO_BARCODE_SEQ\n" ;

                              }


close (FILE);

open (OUTPUT, ">OUTPUT_$FILE_NAME_NO_EXTENSION\_Barcodes_Removed.phylip") or die "Can't open
$file for reading" ;
print OUTPUT "$OUTPUT" ;
close (OUTPUT);
```

```
#####################################          16S C1 alignment.pl
NAME OF INPUT FILE
$INPUT_FILE_NAME = "Yeast_rumen_cultures_3_samples_Q33_FOR_REV_trim_400-600_nt.phylip" ;
open (PHYLIP, "$INPUT_FILE_NAME") or die "Can't open $file for reading" ;

$TEMPLATE = "AGAGTTTGATCATGGCTCAGGAT-GAA-CGCTAG-CTA-CAGG-CTTAA-CAC-ATGC-AAGTCGAGCGGTA-" ;
#for datasets
@ARRAY_NT_TEMPLATE = split('',$TEMPLATE) ; $LENGTH_ARRAY_TEMPLATE = scalar(@ARRAY_NT_TEMPLATE) ;
$LIST_DASH_POSITIONS = "" ; $COUNT_SCREEN_TEMPLATE = 0 ;
print "Determining position of dashes in template\n" ;
while ($COUNT_SCREEN_TEMPLATE < $LENGTH_ARRAY_TEMPLATE) {
        my $NT = $ARRAY_NT_TEMPLATE[$COUNT_SCREEN_TEMPLATE] ;
        if ($NT =~ /-/) {$LIST_DASH_POSITIONS = $LIST_DASH_POSITIONS.="$COUNT_SCREEN_TEMPLATE\t"
;}
        $COUNT_SCREEN_TEMPLATE = $COUNT_SCREEN_TEMPLATE + 1 ;

        }
print "Determining length of sequence blocks in template\n" ;
@ARRAY_BLOCK_TEMPLATE = split('-',$TEMPLATE) ;
$NUMBER_ARRAY_BLOCK = scalar(@ARRAY_BLOCK_TEMPLATE) ;
$LIST_BLOCK_SIZES = "" ; $COUNT_SCREEN_TEMPLATE = 0 ;

while ($COUNT_SCREEN_TEMPLATE < $NUMBER_ARRAY_BLOCK)        {
        my $NT_BLOCK = @ARRAY_BLOCK_TEMPLATE[$COUNT_SCREEN_TEMPLATE] ;
        my $LENGTH_NT_BLOCK = length($NT_BLOCK) ;
        $LIST_BLOCK_SIZES = $LIST_BLOCK_SIZES.="$LENGTH_NT_BLOCK\t" ;
        $COUNT_SCREEN_TEMPLATE = $COUNT_SCREEN_TEMPLATE + 1;

        }


@ARRAY_DASH_POSITIONS = split('\t', $LIST_DASH_POSITIONS) ;
$NUMBER_OF_DASHES = scalar(@ARRAY_DASH_POSITIONS) ;
@ARRAY_BLOCK_SIZES = split('\t', $LIST_BLOCK_SIZES) ;

$READ_COUNT = 0 ; $PRE_OUTPUT = "" ;

while (<PHYLIP>) {
        $READ_COUNT = $READ_COUNT + 1 ; my $COUNT_THOUSAND = $READ_COUNT/2000 ;
        my $COUNT_INTEGER = int($COUNT_THOUSAND) ; my $INTEGER_CHECK = $COUNT_THOUSAND -
$COUNT_INTEGER;
        if ($INTEGER_CHECK == 0) {print "Checking sequence $READ_COUNT\n";  }

        my $LINE = $_ ;        chomp ($LINE); my @ARRAY_LINE = split('\t',$LINE) ;
        my $NAME = $ARRAY_LINE[0] ;   my $SEQ = $ARRAY_LINE[1] ; my $LENGTH_SEQ = length($SEQ) ;

        my $ADJUSTED_SEQ = "" ;
        my $DASH_COUNT = 0 ; my $START = 0; my $DASH_POS = 0 ; my $LENGTH_BLOCK = 0 ;
        while ($DASH_COUNT < $NUMBER_OF_DASHES) {
                $DASH_POS = $ARRAY_DASH_POSITIONS[$DASH_COUNT] ;
                $LENGTH_BLOCK = $ARRAY_BLOCK_SIZES[$DASH_COUNT] ;
                my $BLOCK_SEQ = substr($SEQ, $START, $LENGTH_BLOCK) ;
                $ADJUSTED_SEQ = $ADJUSTED_SEQ.="$BLOCK_SEQ-" ;
                $START = $START + $LENGTH_BLOCK ;
                $DASH_COUNT = $DASH_COUNT + 1;
                                                                                     }
        my $LENGTH_DOWNSTREAM = $LENGTH_SEQ - $START ;
        my $DOWNSTREAM_SEQ = substr($SEQ, $START, $LENGTH_DOWNSTREAM) ;

        $PRE_OUTPUT = $PRE_OUTPUT.="$NAME\t$ADJUSTED_SEQ" ;
        $PRE_OUTPUT = $PRE_OUTPUT.="$DOWNSTREAM_SEQ\n" ;

                              }
close (PHYLIP);


open (OUTPUT, ">OUTPUT_5prime_adjusted_$INPUT_FILE_NAME") or die "Can't open $file for reading" ;
print OUTPUT "$PRE_OUTPUT" ;
close (OUTPUT);
```

```
######################################       16S C1 alignment check.pl
$INPUT_FILE_NAME = " NAME OF INPUT FILE" ;
open (PHYLIP, "$INPUT_FILE_NAME") or die "Can't open $file for reading" ;

$CONSENSUS = "AG.GTTTGATC.TGGCTCAG...-...-......-...-....-.....-...-....-A." ;
$READ_COUNT = 0 ;
$OUTPUT_GOOD = "" ; $OUTPUT__TO_CHECK = "" ;
$GOOD_COUNT = 0 ; $TO_CHECK_COUNT = 0 ;

while (<PHYLIP>) {
        $READ_COUNT = $READ_COUNT + 1 ; my $COUNT_THOUSAND = $READ_COUNT/2000 ;
        my $COUNT_INTEGER = int($COUNT_THOUSAND) ; my $INTEGER_CHECK = $COUNT_THOUSAND -
$COUNT_INTEGER;
        if ($INTEGER_CHECK == 0) {print "Checking sequence $READ_COUNT\n";  }

        my $LINE = $_ ;        chomp ($LINE); my @ARRAY_LINE = split('\t',$LINE) ;
        my $NAME = $ARRAY_LINE[0] ;   my $SEQ = $ARRAY_LINE[1] ; my $LENGTH_SEQ = length($SEQ) ;

        if ($SEQ =~ /$CONSENSUS/)      {
                $OUTPUT_GOOD = $OUTPUT_GOOD.="$NAME\t$SEQ\n" ;
                $GOOD_COUNT = $GOOD_COUNT + 1 ;
                                                             }
        if ($SEQ !~ /$CONSENSUS/)      {
                $OUTPUT__TO_CHECK = $OUTPUT__TO_CHECK.="$NAME\t$SEQ\n" ;
                $TO_CHECK_COUNT = $TO_CHECK_COUNT + 1 ;
                                                             }

                                }
close (PHYLIP);


open (OUTPUT, ">OUTPUT_5prime_checked_sequence_$INPUT_FILE_NAME") or die "Can't open $file for
reading" ;
print OUTPUT "good sequences have consensus $CONSENSUS-:$GOOD_COUNT\n$OUTPUT_GOOD\n" ;
close (OUTPUT);

open (OUTPUT_2, ">OUTPUT_5prime_recheck_sequence_$INPUT_FILE_NAME") or die "Can't open $file for
reading" ;
print OUTPUT_2 "sequences to check:$TO_CHECK_COUNT\n$OUTPUT__TO_CHECK\n" ;
close (OUTPUT_2);
```

```
######################################          16S C2 alignment.pl
$INPUT_FILE_NAME = " NAME OF INPUT FILE" ;
open (PHYLIP, "$INPUT_FILE_NAME") or die "Can't open $file for reading" ;


$V1_TOTAL_LENGTH = 150 ;
$V1_C2_TOTAL_LENGTH = 212 ;

$CONSENSUS_1A = "TAATAC...AT" ;
$CONSENSUS_1B = "TAACAC....T" ;
$CONSENSUS_1C = "TAATAC....C" ;
$CONSENSUS_1D = "T.ATAC...AT" ;
$CONSENSUS_1E = "TAA..CC..GC" ;
$CONSENSUS_1F = "TGATAC...GT" ;
$CONSENSUS_1G = "TAA.AC...AT" ;

$CONSENSUS_2 =  "TAA..CC..AT" ;
$CONSENSUS_3 =  "TAA..CT..AT" ;
$CONSENSUS_4 =  "TAA..CC..CT" ;
$CONSENSUS_5 =  "TAA..CT..CT" ;
$CONSENSUS_6 =  "TAA..CC..AG" ;
$CONSENSUS_7 =  "TAA..CC..CG" ;
$CONSENSUS_8 =  "TAA..CT..AG" ;
$CONSENSUS_9 =  "TAA..CT..CG" ;

$CONSENSUS_10 = "CAAAACCGAAT" ;
$CONSENSUS_11 = "GAGGA.CC.C." ;
$CONSENSUS_12 = "TAATG.C..G." ;
$CONSENSUS_12A ="TAAT..CG.GT" ;
$CONSENSUS_13 = "T.ATC.C...." ; #this one is probaly too loose
$CONSENSUS_13B= "TAA...C..AT" ;
$CONSENSUS_14 = "A.TCCCAATAT" ;


$CONSENSUS_LIST =
"$CONSENSUS_1A,$CONSENSUS_1B,$CONSENSUS_1C,$CONSENSUS_1D,$CONSENSUS_1E,$CONSENSUS_1F,$CONSENSUS_1
G,";
$CONSENSUS_LIST =
$CONSENSUS_LIST.="$CONSENSUS_2,$CONSENSUS_3,$CONSENSUS_4,$CONSENSUS_5,$CONSENSUS_6,$CONSENSUS_7"
;
$CONSENSUS_LIST =
$CONSENSUS_LIST.=",$CONSENSUS_8,$CONSENSUS_9,$CONSENSUS_10,$CONSENSUS_11,$CONSENSUS_12,$CONSENSUS
_12A" ;
$CONSENSUS_LIST = $CONSENSUS_LIST.=",$CONSENSUS_13,$CONSENSUS_13B,$CONSENSUS_14" ;
@ARRAY_CONSENSUS = split(',',$CONSENSUS_LIST) ;
$NUMBER_CONSENSUS_SEQ = scalar(@ARRAY_CONSENSUS) ;

$OUTPUT_FOUND = "" ; $OUTPUT_NOT_FOUND = "" ; $OUTPUT_SUMMARY = "" ;
$OUTPUT_MULTIPLE_CONSENSUS = "" ;
%HASH_COUNT ; $COUNT_HASH_SET_UP = 0 ;
%HASH_POSITION_BASED_ON_DASHES ; $HIGHEST_NUMBER_DASHES = 0 ;
while ($COUNT_HASH_SET_UP < $NUMBER_CONSENSUS_SEQ)   {
        $HASH_COUNT{$COUNT_HASH_SET_UP} = 0 ;
        $COUNT_HASH_SET_UP = $COUNT_HASH_SET_UP + 1 ;

        }
$READ_COUNT = 0 ;
while (<PHYLIP>)       {
        $READ_COUNT = $READ_COUNT + 1 ; my $COUNT_THOUSAND = $READ_COUNT/2000 ;
        my $COUNT_INTEGER = int($COUNT_THOUSAND) ; my $INTEGER_CHECK = $COUNT_THOUSAND -
$COUNT_INTEGER;
        if ($INTEGER_CHECK == 0) {print "Aligning sequence $READ_COUNT\n";  }
#       print "Checking sequence $READ_COUNT\n";
        my $LINE = $_ ;        chomp ($LINE);

        if ($LINE =~ />/) {
                my @ARRAY_LINE = split('\t',$LINE) ;
                my $NAME = $ARRAY_LINE[0] ;   my $SEQ = $ARRAY_LINE[1] ; my $LENGTH_SEQ =
length($SEQ) ;

                my @ARRAY_SEQ_BLOCKS = split('-', $SEQ) ;
```

```
                my $NUMBER_OF_BLOCKS = scalar(@ARRAY_SEQ_BLOCKS) ;
                my $SECOND_TO_LAST_BLOCK = $NUMBER_OF_BLOCKS - 1 ;
                my $LENGTH_FIVE_PRIME = 0 ;
                my $COUNT_BLOCK = 0 ; my $REBUILDING_SEQUENCE = "" ;
                while ($COUNT_BLOCK < $NUMBER_OF_BLOCKS) {
                        my $BLOCK = $ARRAY_SEQ_BLOCKS[$COUNT_BLOCK] ;
                        $REBUILDING_SEQUENCE = $REBUILDING_SEQUENCE.="$BLOCK-" ;
                        if ($COUNT_BLOCK < $SECOND_TO_LAST_BLOCK)    {
                                $LENGTH_FIVE_PRIME = length($REBUILDING_SEQUENCE) ;

        }
                        $COUNT_BLOCK = $COUNT_BLOCK + 1 ;
                                                                                        }
                my $DOWNSTREAM_REGION = $ARRAY_SEQ_BLOCKS[$COUNT_BLOCK] ;
                my $CONSENSUS_FOUND = 0 ; my $CONSENSUS_FOUND_COPY = 0 ;
                my $ALIGNED_SEQ = "" ; my $NUMBER_OF_DASHES = 0 ;
                my $COUNT_SCREEN = 0 ;
                while ($COUNT_SCREEN < $NUMBER_CONSENSUS_SEQ and $CONSENSUS_FOUND < 1)     {
                        my $CONSENSUS_TO_SCREEN = $ARRAY_CONSENSUS[$COUNT_SCREEN] ;
                        my $LENGTH_CONSENSUS = length($CONSENSUS_TO_SCREEN) ;
                        if ($SEQ =~ /$CONSENSUS_TO_SCREEN/)   {
                                $CONSENSUS_FOUND = 2 ;
                                my $INFO_TO_UPDATE = $HASH_COUNT{$COUNT_SCREEN} ;
                                $INFO_TO_UPDATE = $INFO_TO_UPDATE + 1 ;
                                $HASH_COUNT{$COUNT_SCREEN} = $INFO_TO_UPDATE ;

                                my $FIVE_PRIME_BOUNDARY =  0 ; my $THREE_PRIME_BOUNDARY = 0 ;
                                my $DOWNSTREAM_LENGTH = 0 ;
                                my $THREE_PRIME_FOUND =  0 ;
                                my $LENGTH_TO_SCREEN = $LENGTH_CONSENSUS ;
                                while ($THREE_PRIME_FOUND < 1)       {
                                        my $DOWNSTREAM_SEARCH = substr($SEQ, 0, $LENGTH_TO_SCREEN)
;
                                        if ($DOWNSTREAM_SEARCH !~ /$CONSENSUS_TO_SCREEN/)    {
                                                $LENGTH_TO_SCREEN = $LENGTH_TO_SCREEN + 1 ;

                                }
                                        if ($DOWNSTREAM_SEARCH =~ /$CONSENSUS_TO_SCREEN/)    {
                                                $FIVE_PRIME_BOUNDARY = $LENGTH_TO_SCREEN -
$LENGTH_CONSENSUS;
                                                $THREE_PRIME_BOUNDARY = $LENGTH_TO_SCREEN ;
                                                $DOWNSTREAM_LENGTH = $LENGTH_SEQ -
$THREE_PRIME_BOUNDARY ;
                                                $THREE_PRIME_FOUND =  2 ;

                                        }

                                                                                        }
                                my $FIVE_PRIME_REGION = substr($SEQ, 0, $LENGTH_FIVE_PRIME) ;
#                               my $CONSERVED_REGION = substr($SEQ,$FIVE_PRIME_BOUNDARY,
$LENGTH_CONSENSUS) ;
                                my $LENGTH_V1_C2_NOT_ALIGNED = $THREE_PRIME_BOUNDARY -
$LENGTH_FIVE_PRIME ;
                                my $V1_C2 = substr($SEQ,$LENGTH_FIVE_PRIME,
$LENGTH_V1_C2_NOT_ALIGNED);
                                $NUMBER_OF_DASHES = $V1_C2_TOTAL_LENGTH - $LENGTH_V1_C2_NOT_ALIGNED
;
                                my $DASHES_COUNT = 0 ; my $V1_DASHES = "" ;
                                while ($DASHES_COUNT < $NUMBER_OF_DASHES)    {
                                        $V1_DASHES = $V1_DASHES.="-" ;
                                        $DASHES_COUNT = $DASHES_COUNT + 1 ;

                }
                                my $DOWNSTREAM = substr($SEQ,$THREE_PRIME_BOUNDARY
,$DOWNSTREAM_LENGTH) ;
                                $ALIGNED_SEQ = "$FIVE_PRIME_REGION-$V1_DASHES-$V1_C2--$DOWNSTREAM"
;
                                #$OUTPUT_FOUND = $OUTPUT_FOUND.="$NAME\t$ALIGNED_SEQ\n" ;
                                my $SEQ_HASH_TRANSFER =
$HASH_POSITION_BASED_ON_DASHES{$NUMBER_OF_DASHES} ;
                                $SEQ_HASH_TRANSFER = $SEQ_HASH_TRANSFER.="$NAME\t$ALIGNED_SEQ\n" ;
```

```
                              $HASH_POSITION_BASED_ON_DASHES{$NUMBER_OF_DASHES} =
$SEQ_HASH_TRANSFER ;
                              if ($NUMBER_OF_DASHES > $HIGHEST_NUMBER_DASHES )     {
                                     $HIGHEST_NUMBER_DASHES = $NUMBER_OF_DASHES ;

                              }
#determining if other copies of consensus present downstream
                              my $COUNT_SCREEN_COPY = 0 ;
                              while ($COUNT_SCREEN_COPY < $NUMBER_CONSENSUS_SEQ and
$CONSENSUS_FOUND_COPY < 1)    {
                                     my $CONSENSUS_TO_SCREEN =
$ARRAY_CONSENSUS[$COUNT_SCREEN_COPY] ;
                                     my $LENGTH_CONSENSUS = length($CONSENSUS_TO_SCREEN) ;
                                     if ($DOWNSTREAM  =~ /$CONSENSUS_TO_SCREEN/)  {
$CONSENSUS_FOUND_COPY = 2 ; }
                                     $COUNT_SCREEN_COPY = $COUNT_SCREEN_COPY + 1 ;

                                                                                    }

                                            }
                       $COUNT_SCREEN = $COUNT_SCREEN + 1 ;

                                                   }
               if ($CONSENSUS_FOUND < 1)     {
                       $OUTPUT_NOT_FOUND = $OUTPUT_NOT_FOUND.="$LINE\n" ;
                                                                  }
               if ($CONSENSUS_FOUND_COPY > 1)       {
                       $OUTPUT_MULTIPLE_CONSENSUS = $OUTPUT_MULTIPLE_CONSENSUS.="$LINE\n" ;
                                                                         }
               if ($CONSENSUS_FOUND_COPY < 1)       {

                                                                         }
                                            }
                                     }
close (PHYLIP);

print "Preparing frequency output\n" ;
$COUNT_OUTPUT = 0;
while ($COUNT_OUTPUT < $NUMBER_CONSENSUS_SEQ)       {
       my $CURRENT_CONSENSUS = $ARRAY_CONSENSUS[$COUNT_OUTPUT] ;
       my $FREQUENCY = $HASH_COUNT{$COUNT_OUTPUT} ;
       $OUTPUT_SUMMARY = $OUTPUT_SUMMARY.="Frequency of $CURRENT_CONSENSUS:\t$FREQUENCY\n" ;
       $COUNT_OUTPUT = $COUNT_OUTPUT + 1 ;
                                                                                    }

print "Preparing output\n" ;
$COUNT_OUTPUT_2 = $HIGHEST_NUMBER_DASHES ;
while ($COUNT_OUTPUT_2 > 0)   {
       my $INFO_TO_OUTPUT = $HASH_POSITION_BASED_ON_DASHES{$COUNT_OUTPUT_2} ;
       chomp ($INFO_TO_OUTPUT) ;
       my $SIZE_INFO_TO_OUTPUT = length($INFO_TO_OUTPUT) ;
       if ($SIZE_INFO_TO_OUTPUT > 0)        {
               $OUTPUT_FOUND = $OUTPUT_FOUND.="$INFO_TO_OUTPUT\n" ;
                                                                  }
       $COUNT_OUTPUT_2 = $COUNT_OUTPUT_2 - 1 ;
                                                          }

open (OUTPUT, ">OUTPUT_C2_region_adjusted_$INPUT_FILE_NAME") or die "Can't open $file for
reading" ;
print OUTPUT "$OUTPUT_SUMMARY" ;
print OUTPUT "$OUTPUT_FOUND";
close (OUTPUT);

open (OUTPUT_2, ">OUTPUT_C2_region_MULTIPLE_copies_$INPUT_FILE_NAME") or die "Can't open $file
for reading" ;
print OUTPUT_2 "Sequences with multiple copies of consensus\n" ;
print OUTPUT_2 "$OUTPUT_MULTIPLE_CONSENSUS\n" ;
close (OUTPUT_2);

open (OUTPUT_3, ">OUTPUT_C2_region_NOT_FOUND_$INPUT_FILE_NAME") or die "Can't open $file for
reading" ;
```

```
print OUTPUT_3 "Sequences with no copies of consensus\n" ;
print OUTPUT_3 "$OUTPUT_NOT_FOUND";
close (OUTPUT_3) ;
```

```
######################################       16S C2 alignment check.pl
$INPUT_FILE_NAME = " NAME OF INPUT FILE" ;
open (PHYLIP, "$INPUT_FILE_NAME") or die "Can't open $file for reading" ;

$POSITION_TO_CHECK = 287 ;
$LENGTH_TO_CHECK = 2 ;
$PATTERN =  "--" ;
$LENGTH_PATTERN = length($PATTERN) ;


$OUTPUT_OK = "" ; $OUTPUT_NOT_OK = "" ;

while (<PHYLIP>)       {
       $READ_COUNT = $READ_COUNT + 1 ; my $COUNT_THOUSAND = $READ_COUNT/2000 ;
       my $COUNT_INTEGER = int($COUNT_THOUSAND) ; my $INTEGER_CHECK = $COUNT_THOUSAND - 
$COUNT_INTEGER;
       if ($INTEGER_CHECK == 0) {print "Checking sequence $READ_COUNT\n";  }

       my $LINE = $_ ;        chomp ($LINE);
       if ($LINE =~ />/)      {
              my @ARRAY_LINE = split('\t',$LINE) ;
              my $NAME = $ARRAY_LINE[0] ;   my $SEQ = $ARRAY_LINE[1] ; my $LENGTH_SEQ = 
length($SEQ) ;

              my $REGION_TO_CHECK = substr($SEQ,$POSITION_TO_CHECK,$LENGTH_TO_CHECK) ;
              if ($REGION_TO_CHECK ne $PATTERN) {$OUTPUT_NOT_OK = $OUTPUT_NOT_OK.="$LINE\n" ; }
              if ($REGION_TO_CHECK eq $PATTERN)     {
                     my $SECOND_CHECK = 0 ;
                     my $UPSTREAM_POSITION = $POSITION_TO_CHECK - 1 ;
                     my $UPSTREAM_TO_CHECK = substr($SEQ,$UPSTREAM_POSITION,1) ;
                     if ($UPSTREAM_TO_CHECK eq "-") {$SECOND_CHECK = 2 ;}

                     my $DOWNSTREAM_POSITION = $POSITION_TO_CHECK + $LENGTH_PATTERN ;
                     my $DOWNSTREAM_TO_CHECK = substr($SEQ,$DOWNSTREAM_POSITION,1) ;
                     if ($DOWNSTREAM_TO_CHECK eq "-") {$SECOND_CHECK = 2 ;}

                     if ($SECOND_CHECK < 1)        {
                            $OUTPUT_OK = $OUTPUT_OK.="$LINE\n" ;
                                                                    }
                     if ($SECOND_CHECK > 1)        {
                            $OUTPUT_NOT_OK = $OUTPUT_NOT_OK.="$LINE\n" ;
                                                                    }
                                                                                  }
                                          }
                                   }
close (PHYLIP);

print "Preparing frequency output\n" ;
$COUNT_OUTPUT = 0;
while ($COUNT_OUTPUT < $NUMBER_CONSENSUS_SEQ)        {
       my $CURRENT_CONSENSUS = $ARRAY_CONSENSUS[$COUNT_OUTPUT] ;
       my $FREQUENCY = $HASH_COUNT{$COUNT_OUTPUT} ;
       $OUTPUT_SUMMARY = $OUTPUT_SUMMARY.="Frequency of $CURRENT_CONSENSUS:\t$FREQUENCY\n" ;
       $COUNT_OUTPUT = $COUNT_OUTPUT + 1 ;
                                                                                  }

open (OUTPUT, ">OUTPUT_C2_region_confirmed_$INPUT_FILE_NAME") or die "Can't open $file for 
reading" ;
print OUTPUT "$OUTPUT_OK";
close (OUTPUT);

open (OUTPUT_2, ">OUTPUT_C2_region_to_check_$INPUT_FILE_NAME") or die "Can't open $file for 
reading" ;
print OUTPUT_2 "$OUTPUT_NOT_OK";
close (OUTPUT_2);
```

```
######################################          16S C3 begin alignment.pl
$INPUT_FILE_NAME = " NAME OF INPUT FILE" ;
open (PHYLIP, "$INPUT_FILE_NAME") or die "Can't open $file for reading" ;

$V2_TOTAL_LENGTH = 150 ;
$CONSENSUS_1A =  ".CG....ATTAG.T.GTTGGT......" ;            #C3a begin
$CONSENSUS_1B =  ".CG....GTTAG.T.GTTGGC......";             #other 03102019
$CONSENSUS_2 =  ".CG....ATTAG.T.GT.GG.....TA" ;      #C3b begin
$CONSENSUS_3 =  "A.A....ATTAG.T.GT.GG.....TA" ;      #C3c begin
$CONSENSUS_4 =  "G.G....ATTAG...G..GG.....C.";       #C3d begin
$CONSENSUS_5 =  "GC.....AT.AG.T.GTTGG.....TA" ; #BSTP
$CONSENSUS_21 = ".......AT.AG.T.GTTGG.....TA" ;      #BSTP
$CONSENSUS_22 = "G......ATTA....G..GG......A" ;          #GCGTCTGATTAACTAGTTGGTAAGGTA
$CONSENSUS_23 = "G......A.TAG...G..GG......A" ;          #GCGTTCCAGTAGCTAGTTGGTGAGGTA
$CONSENSUS_24 = "...........G.T.GT.G......TA";
$CONSENSUS_25 = "GCG.........T......T...GTA";
$CONSENSUS_26 = ".......AT......GTTGG.....TA";
$CONSENSUS_27 = "GC.....ATTAG.T.G....T....TA";

$CONSENSUS_31= "GCG.........T.........GTA" ;
$CONSENSUS_32= "G.G.........T.........GTA" ;
$CONSENSUS_33= ".CG.........T..........TA" ;
$CONSENSUS_34= ".GG.........T.G..GG.....CA" ;
$CONSENSUS_35= ".G..........T.G..G......CA" ;

$CONSENSUS_LAST1 = ".......ATTAG...G..GG.......";
$CONSENSUS_LAST2 = ".......AT.AG...G..GG.......";

$LIST_CONSENSUS =
"$CONSENSUS_1A,$CONSENSUS_1B,$CONSENSUS_2,$CONSENSUS_3,$CONSENSUS_4,$CONSENSUS_5" ;
$LIST_CONSENSUS =
$LIST_CONSENSUS.=",$CONSENSUS_21,$CONSENSUS_22,$CONSENSUS_23,$CONSENSUS_24,$CONSENSUS_25" ;
$LIST_CONSENSUS = $LIST_CONSENSUS.=",$CONSENSUS_26,$CONSENSUS_27" ;
$LIST_CONSENSUS = $LIST_CONSENSUS.=",$CONSENSUS_LAST1,$CONSENSUS_LAST2" ;
$LIST_CONSENSUS =
$LIST_CONSENSUS.=",$CONSENSUS_31,$CONSENSUS_32,$CONSENSUS_33,$CONSENSUS_34,$CONSENSUS_35" ;
@ARRAY_LIST = split(',', $LIST_CONSENSUS) ; $NUMBER_CONSENSUS = scalar(@ARRAY_LIST) ;

%HASH_COUNT ; $COUNT_HASH_SET_UP = 0 ;
while ($COUNT_HASH_SET_UP < $NUMBER_CONSENSUS)       {
        $HASH_COUNT{$COUNT_HASH_SET_UP} = 0 ;
        $COUNT_HASH_SET_UP = $COUNT_HASH_SET_UP + 1 ;
                                                                                                    }

$OUTPUT_NOT_FOUND = "" ; $OUTPUT_FOUND = "" ; $OUTPUT_SUMMARY = "" ;
$READ_COUNT = 0 ; $COUNT_NOT_FOUND = 0 ;
while (<PHYLIP>)       {
        $READ_COUNT = $READ_COUNT + 1 ; my $COUNT_THOUSAND = $READ_COUNT/2000 ;
        my $COUNT_INTEGER = int($COUNT_THOUSAND) ; my $INTEGER_CHECK = $COUNT_THOUSAND -
$COUNT_INTEGER;
        if ($INTEGER_CHECK == 0) {print "Checking sequence $READ_COUNT\n";  }

        my $LINE = $_ ;        chomp ($LINE); my @ARRAY_LINE = split('\t',$LINE) ;
        my $NAME = $ARRAY_LINE[0] ;   my $SEQ = $ARRAY_LINE[1] ; my $LENGTH_SEQ = length($SEQ) ;

        my @ARRAY_SEQ_BLOCKS = split('-', $SEQ) ;
        my $NUMBER_OF_BLOCKS = scalar(@ARRAY_SEQ_BLOCKS) ;
        my $SECOND_TO_LAST_BLOCK = $NUMBER_OF_BLOCKS - 1 ;
        my $LENGTH_FIVE_PRIME = 0 ;
        my $COUNT_BLOCK = 0 ; my $REBUILDING_SEQUENCE = "" ;
        while ($COUNT_BLOCK < $SECOND_TO_LAST_BLOCK)        {
                my $BLOCK = $ARRAY_SEQ_BLOCKS[$COUNT_BLOCK] ;
                $REBUILDING_SEQUENCE = $REBUILDING_SEQUENCE.="$BLOCK-" ;
                $COUNT_BLOCK = $COUNT_BLOCK + 1 ;

        }
        my $LENGTH_FIVE_PRIME = length($REBUILDING_SEQUENCE) ;
        my $DOWNSTREAM_REGION_TO_SCREEN = $ARRAY_SEQ_BLOCKS[$SECOND_TO_LAST_BLOCK ] ;
        my $LENGTH_DOWNSTREAM_TO_SCREEN = length($DOWNSTREAM_REGION_TO_SCREEN) ;
        my $COUNT_ARRAY = 0 ; my $CONSENSUS_FOUND = 0 ;
        while ($COUNT_ARRAY < $NUMBER_CONSENSUS and $CONSENSUS_FOUND < 1)   {
```

```
		my $CURRENT_CONSENSUS = $ARRAY_LIST[$COUNT_ARRAY] ;
		my $LENGTH_CONSENSUS = length($CURRENT_CONSENSUS) ;
		if ($DOWNSTREAM_REGION_TO_SCREEN =~ /$CURRENT_CONSENSUS/)	{
			$CONSENSUS_FOUND = 2 ;
			my $INFO_TO_UPDATE = $HASH_COUNT{$COUNT_ARRAY} ;
			$INFO_TO_UPDATE = $INFO_TO_UPDATE + 1 ;
			$HASH_COUNT{$COUNT_ARRAY} = $INFO_TO_UPDATE ;


			my $FIVE_PRIME_BOUNDARY =  0 ; my $THREE_PRIME_BOUNDARY = 0 ;
			my $DOWNSTREAM_LENGTH = 0 ; my $THREE_PRIME_FOUND =  0 ;
			my $LENGTH_TO_SCREEN = length($CURRENT_CONSENSUS) ;
			while ($THREE_PRIME_FOUND < 1)		{
				my $DOWNSTREAM_SEARCH = substr($DOWNSTREAM_REGION_TO_SCREEN, 0,
$LENGTH_TO_SCREEN) ;
				if ($DOWNSTREAM_SEARCH !~ /$CURRENT_CONSENSUS/)		{
					$LENGTH_TO_SCREEN = $LENGTH_TO_SCREEN + 1 ;

			}
				if ($DOWNSTREAM_SEARCH =~ /$CURRENT_CONSENSUS/)		{
					$FIVE_PRIME_BOUNDARY = $LENGTH_TO_SCREEN -
$LENGTH_CONSENSUS;
					$THREE_PRIME_BOUNDARY = $LENGTH_TO_SCREEN ;
					$DOWNSTREAM_LENGTH = $LENGTH_DOWNSTREAM_TO_SCREEN -
$THREE_PRIME_BOUNDARY ;
					$THREE_PRIME_FOUND =  2 ;

			}

								}
			my $FIVE_PRIME_REGION = substr($SEQ, 0, $LENGTH_FIVE_PRIME) ;
			my $CONSERVED_REGION =
substr($DOWNSTREAM_REGION_TO_SCREEN,$FIVE_PRIME_BOUNDARY, $LENGTH_CONSENSUS) ;
#			$OUTPUT_FOUND = $OUTPUT_FOUND.="$CONSERVED_REGION\n" ;
#			my $LENGTH_V2_NOT_ALIGNED = $FIVE_PRIME_BOUNDARY - $LENGTH_FIVE_PRIME ;
			my $LENGTH_V2_NOT_ALIGNED = $FIVE_PRIME_BOUNDARY ;
			my $V2 = substr($DOWNSTREAM_REGION_TO_SCREEN,0, $LENGTH_V2_NOT_ALIGNED);
			my $NUMBER_OF_DASHES = $V2_TOTAL_LENGTH - $LENGTH_V2_NOT_ALIGNED ;
			my $DASHES_COUNT = 0 ; my $V2_DASHES = "" ;
			while ($DASHES_COUNT < $NUMBER_OF_DASHES)	{
				$V2_DASHES = $V2_DASHES.="-" ;
				$DASHES_COUNT = $DASHES_COUNT + 1 ;

	}
			my $DOWNSTREAM = substr($DOWNSTREAM_REGION_TO_SCREEN,$THREE_PRIME_BOUNDARY
,$DOWNSTREAM_LENGTH) ;
			my $ALIGNED_SEQ = "$FIVE_PRIME_REGION-$V2_DASHES-$V2-$CONSERVED_REGION-
$DOWNSTREAM"  ;
#			$OUTPUT_FOUND = $OUTPUT_FOUND.="$LINE\n" ;
			$OUTPUT_FOUND = $OUTPUT_FOUND.="$NAME\t$ALIGNED_SEQ\n" ;

				}
		$COUNT_ARRAY = $COUNT_ARRAY + 1 ;

					}

	if ($CONSENSUS_FOUND < 1)	{
#		$COUNT_NOT_FOUND = $COUNT_NOT_FOUND + 1 ;
		$OUTPUT_NOT_FOUND = $OUTPUT_NOT_FOUND."$LINE\n" ;
							}
				}
close (PHYLIP);
#####################################
print "Preparing frequency output\n" ;
$COUNT_OUTPUT = 0;
while ($COUNT_OUTPUT < $NUMBER_CONSENSUS)	{
	my $CURRENT_CONSENSUS = $ARRAY_LIST[$COUNT_OUTPUT] ;
	my $FREQUENCY = $HASH_COUNT{$COUNT_OUTPUT} ;
	$OUTPUT_SUMMARY = $OUTPUT_SUMMARY.="Frequency of $CURRENT_CONSENSUS:\t$FREQUENCY\n" ;
	$COUNT_OUTPUT = $COUNT_OUTPUT + 1 ;
								}
```

```
open (OUTPUT, ">OUTPUT_C3_begin_adjusted_$INPUT_FILE_NAME") or die "Can't open $file for reading"
;
print OUTPUT "$OUTPUT_SUMMARY" ;
print OUTPUT "$OUTPUT_FOUND";
close (OUTPUT);

open (OUTPUT_2, ">OUTPUT_C3_begin_NOT_FOUND_$INPUT_FILE_NAME") or die "Can't open $file for
reading" ;
print OUTPUT_2 "$OUTPUT_NOT_FOUND";
close (OUTPUT_2);
```

```
######################################        16S C3 begin alignment check.pl
$INPUT_FILE_NAME = " NAME OF INPUT FILE" ;
open (PHYLIP, "$INPUT_FILE_NAME") or die "Can't open $file for reading" ;

$POSITION_TO_CHECK = 441 ;
$LENGTH_TO_CHECK = 29 ;
$PATTERN_TEST =  "-GCGGTGCATTAGTTAGTTGGTGGGGTA-" ;
$PATTERN =       "-...........................-" ;

$OUTPUT_OK = "" ; $OUTPUT_NOT_OK = "" ;


while (<PHYLIP>)       {
       $READ_COUNT = $READ_COUNT + 1 ; my $COUNT_THOUSAND = $READ_COUNT/2000 ;
       my $COUNT_INTEGER = int($COUNT_THOUSAND) ; my $INTEGER_CHECK = $COUNT_THOUSAND -
$COUNT_INTEGER;
       if ($INTEGER_CHECK == 0) {print "Checking sequence $READ_COUNT\n";  }

       my $LINE = $_ ;        chomp ($LINE); my @ARRAY_LINE = split('\t',$LINE) ;
       my $NAME = $ARRAY_LINE[0] ;   my $SEQ = $ARRAY_LINE[1] ; my $LENGTH_SEQ = length($SEQ) ;

       my $REGION_TO_CHECK = substr($SEQ,$POSITION_TO_CHECK,$LENGTH_TO_CHECK) ;
       if ($REGION_TO_CHECK !~ /$PATTERN/) {$OUTPUT_NOT_OK = $OUTPUT_NOT_OK.="$LINE\n" ; }
       if ($REGION_TO_CHECK =~ /$PATTERN/)   {
              my $DOWNSTREAM_START = $POSITION_TO_CHECK + $LENGTH_TO_CHECK ;
              my $DOWNSTREAM_LENGTH = $LENGTH_SEQ - $DOWNSTREAM_START ;
              my $DOWNSTREAM_REGION = substr($SEQ,$DOWNSTREAM_START,$DOWNSTREAM_LENGTH) ;
              if ($DOWNSTREAM_REGION =~ /-/)        {
                     $OUTPUT_NOT_OK = $OUTPUT_NOT_OK.="$LINE\n" ;
                                                                          }
              if ($DOWNSTREAM_REGION !~ /-/)               {

                     $OUTPUT_OK = $OUTPUT_OK.="$LINE\n" ;
                                                                                 }
                                                                          }
                                    }
close (PHYLIP);

print "Preparing frequency output\n" ;
$COUNT_OUTPUT = 0;
while ($COUNT_OUTPUT < $NUMBER_CONSENSUS_SEQ)        {
       my $CURRENT_CONSENSUS = $ARRAY_CONSENSUS[$COUNT_OUTPUT] ;
       my $FREQUENCY = $HASH_COUNT{$COUNT_OUTPUT} ;
       $OUTPUT_SUMMARY = $OUTPUT_SUMMARY.="Frequency of $CURRENT_CONSENSUS:\t$FREQUENCY\n" ;
       $COUNT_OUTPUT = $COUNT_OUTPUT + 1 ;
                                                                                 }

open (OUTPUT, ">OUTPUT_C3_begin_confirmed_$INPUT_FILE_NAME") or die "Can't open $file for
reading" ;
print OUTPUT "$OUTPUT_OK";
close (OUTPUT);

open (OUTPUT_2, ">OUTPUT_C3_begin_to_check_$INPUT_FILE_NAME") or die "Can't open $file for
reading" ;
print OUTPUT_2 "$OUTPUT_NOT_OK";
close (OUTPUT_2);
```

```
#####################################        16S C3 end alignment.pl
NAME OF INPUT FILE
$INPUT_FILE_NAME = " NAME OF INPUT FILE" ;
open (PHYLIP, "$INPUT_FILE_NAME") or die "Can't open $file for reading" ;

$CONSENSUS_1 =  "TTGTAAA....." ;
$CONSENSUS_2a = ".TGTAAA....." ;
$CONSENSUS_2 =  "T.GTAAA....." ;
$CONSENSUS_3 =  "T..TAAA....." ; #MODIFIED ON 09052018
$CONSENSUS_4 =  "TTG.AAA....." ;
$CONSENSUS_5 =  "TTGT.AA....." ;
$CONSENSUS_6 =  "TTGTA.A....." ;
$CONSENSUS_7 =  "TTGTAA......" ;
$CONSENSUS_8 =  "ACGTAAA....." ;
$CONSENSUS_9 =  ".CGTAAA....." ;
$CONSENSUS_10 = "A.GTAAA....." ;
$CONSENSUS_11 = "AC.TAAA....." ;
$CONSENSUS_12 = "ACG.AAA....." ;
$CONSENSUS_13 = "ACGT.AA....." ;
$CONSENSUS_14 = "ACGTA.A....." ;
$CONSENSUS_15 = "ACGTAA......" ;
$CONSENSUS_16 = "TCGTAAG....." ; #latest addition tested 09052018
$CONSENSUS_17 = "TCTTAAA....." ; #latest addition tested 09052018
$LIST_CONSENSUS =
"$CONSENSUS_1,$CONSENSUS_2,$CONSENSUS_2a,$CONSENSUS_3,$CONSENSUS_4,$CONSENSUS_5,$CONSENSUS_6,$CON
SENSUS_7,$CONSENSUS_8" ;
$LIST_CONSENSUS =
$LIST_CONSENSUS.=",$CONSENSUS_9,$CONSENSUS_10,$CONSENSUS_11,$CONSENSUS_12,$CONSENSUS_13,$CONSENSU
S_14,$CONSENSUS_15" ;
$LIST_CONSENSUS = $LIST_CONSENSUS.=",$CONSENSUS_16,$CONSENSUS_17" ; #latest addition tested
09052018
@ARRAY_LIST = split(',', $LIST_CONSENSUS) ; $NUMBER_CONSENSUS = scalar(@ARRAY_LIST) ;

%HASH_COUNT ; $COUNT_HASH_SET_UP = 0 ;
while ($COUNT_HASH_SET_UP < $NUMBER_CONSENSUS)       {
        $HASH_COUNT{$COUNT_HASH_SET_UP} = 0 ;
        $COUNT_HASH_SET_UP = $COUNT_HASH_SET_UP + 1 ;
                                                                                                }

$OUTPUT_NOT_FOUND = "" ; $OUTPUT_FOUND = "" ; $OUTPUT_SUMMARY = "" ;
$READ_COUNT = 0 ; $COUNT_NOT_FOUND = 0 ;

while (<PHYLIP>)       {
        $READ_COUNT = $READ_COUNT + 1 ; my $COUNT_THOUSAND = $READ_COUNT/2000 ;
        my $COUNT_INTEGER = int($COUNT_THOUSAND) ; my $INTEGER_CHECK = $COUNT_THOUSAND -
$COUNT_INTEGER;
        if ($INTEGER_CHECK == 0) {print "Checking sequence $READ_COUNT\n";  }

        my $LINE = $_ ;        chomp ($LINE); my @ARRAY_LINE = split('\t',$LINE) ;
        my $NAME = $ARRAY_LINE[0] ;   my $SEQ = $ARRAY_LINE[1] ; my $LENGTH_SEQ = length($SEQ) ;

        my @ARRAY_SEQ_BLOCKS = split('-', $SEQ) ;
        my $NUMBER_OF_BLOCKS = scalar(@ARRAY_SEQ_BLOCKS) ;
        my $LAST_BLOCK = $NUMBER_OF_BLOCKS - 1 ;
        my $LENGTH_FIVE_PRIME = 0 ;
        my $COUNT_BLOCK = 0 ; my $REBUILDING_SEQUENCE = "" ;
        while ($COUNT_BLOCK < $LAST_BLOCK) {
                my $BLOCK = $ARRAY_SEQ_BLOCKS[$COUNT_BLOCK] ;
                $REBUILDING_SEQUENCE = $REBUILDING_SEQUENCE.="$BLOCK-" ;
                $COUNT_BLOCK = $COUNT_BLOCK + 1 ;
                                                                                                }
        my $LENGTH_FIVE_PRIME = length($REBUILDING_SEQUENCE) ;
        my $DOWNSTREAM_REGION_TO_SCREEN = $ARRAY_SEQ_BLOCKS[$LAST_BLOCK ] ;
        my $LENGTH_DOWNSTREAM_TO_SCREEN = length($DOWNSTREAM_REGION_TO_SCREEN) ;
        my $COUNT_ARRAY = 0 ; my $CONSENSUS_FOUND = 0 ;
        while ($COUNT_ARRAY < $NUMBER_CONSENSUS and $CONSENSUS_FOUND < 1)   {
                my $CURRENT_CONSENSUS = $ARRAY_LIST[$COUNT_ARRAY] ;
                my $LENGTH_CONSENSUS = length($CURRENT_CONSENSUS) ;
                if ($DOWNSTREAM_REGION_TO_SCREEN =~ /$CURRENT_CONSENSUS/)   {
                        $CONSENSUS_FOUND = 2 ;
                        my $INFO_TO_UPDATE = $HASH_COUNT{$COUNT_ARRAY} ;
```

```
                        $INFO_TO_UPDATE = $INFO_TO_UPDATE + 1 ;
                        $HASH_COUNT{$COUNT_ARRAY} = $INFO_TO_UPDATE ;


                        my $FIVE_PRIME_BOUNDARY =  0 ; my $THREE_PRIME_BOUNDARY = 0 ;
                        my $THREE_PRIME_LENGTH = 0 ; my $THREE_PRIME_FOUND =  0 ;
                        my $LENGTH_TO_SCREEN = length($CURRENT_CONSENSUS) ;
                        while ($THREE_PRIME_FOUND < 1)        {
                                my $DOWNSTREAM_SEARCH = substr($DOWNSTREAM_REGION_TO_SCREEN, 0,
$LENGTH_TO_SCREEN) ;
                                if ($DOWNSTREAM_SEARCH !~ /$CURRENT_CONSENSUS/)      {
                                        $LENGTH_TO_SCREEN = $LENGTH_TO_SCREEN + 1 ;

                        }
                                if ($DOWNSTREAM_SEARCH =~ /$CURRENT_CONSENSUS/)      {
                                        $FIVE_PRIME_BOUNDARY = $LENGTH_TO_SCREEN -
$LENGTH_CONSENSUS;
                                        $THREE_PRIME_BOUNDARY = $LENGTH_TO_SCREEN ;
                                        $THREE_PRIME_LENGTH = $LENGTH_DOWNSTREAM_TO_SCREEN -
$THREE_PRIME_BOUNDARY ;
                                        $THREE_PRIME_FOUND =  2 ;

                        }

                                                                                    }
                        my $FIVE_PRIME_REGION = substr($SEQ, 0, $LENGTH_FIVE_PRIME) ;
                        my $C3 = substr($DOWNSTREAM_REGION_TO_SCREEN,0, $LENGTH_TO_SCREEN) ;
                        my $DOWNSTREAM = substr($DOWNSTREAM_REGION_TO_SCREEN,$THREE_PRIME_BOUNDARY
,$THREE_PRIME_LENGTH) ;
                        my $ALIGNED_SEQ = "$FIVE_PRIME_REGION-$C3-$DOWNSTREAM"  ;
#                       $OUTPUT_FOUND = $OUTPUT_FOUND.="$LINE\n" ; ##FOR CHECKING ON SCRIPT
ACCURACY
                        $OUTPUT_FOUND = $OUTPUT_FOUND.="$NAME\t$ALIGNED_SEQ\n" ;

                                }
                $COUNT_ARRAY = $COUNT_ARRAY + 1 ;


                                        }
        if ($CONSENSUS_FOUND < 1)       {
#               $COUNT_NOT_FOUND = $COUNT_NOT_FOUND + 1 ;
                $OUTPUT_NOT_FOUND = $OUTPUT_NOT_FOUND."$LINE\n" ;
                                                        }
                                        }
close (PHYLIP);

print "Preparing frequency output\n" ;
$COUNT_OUTPUT = 0;
while ($COUNT_OUTPUT < $NUMBER_CONSENSUS)    {
        my $CURRENT_CONSENSUS = $ARRAY_LIST[$COUNT_OUTPUT] ;
        my $FREQUENCY = $HASH_COUNT{$COUNT_OUTPUT} ;
        $OUTPUT_SUMMARY = $OUTPUT_SUMMARY.="Frequency of $CURRENT_CONSENSUS:\t$FREQUENCY\n" ;
        $COUNT_OUTPUT = $COUNT_OUTPUT + 1 ;
                                                                                    }

open (OUTPUT, ">OUTPUT_C3_end_position_adjusted_$INPUT_FILE_NAME") or die "Can't open $file for
reading" ;
print OUTPUT "$OUTPUT_SUMMARY" ;
print OUTPUT "$OUTPUT_FOUND";
close (OUTPUT);

open (OUTPUT_2, ">OUTPUT_C3_end_position_NOT_FOUND_$INPUT_FILE_NAME") or die "Can't open $file
for reading" ;
print OUTPUT_2 "$OUTPUT_NOT_FOUND";
close (OUTPUT_2);
```

```
#####################################          16S C4 alignment.pl
NAME OF INPUT FILE
$INPUT_FILE_NAME = "Yeast_rumen_cultures_3_samples_C3_end_position_adjusted.phylip" ;
open (PHYLIP, "$INPUT_FILE_NAME") or die "Can't open $file for reading" ;

$V3_TOTAL_LENGTH = 150 ;
$CONSENSUS_1A = "CGGCTAA.T" ; $CONSENSUS_1B = ".GGCTAA.T" ; $CONSENSUS_1C = "C.GCTAA.T" ;
$CONSENSUS_1D = "CG.CTAA.T"; $CONSENSUS_1E= "CGG.TAA.T"; $CONSENSUS_1F = "CGGC.AA.T" ;
$CONSENSUS_1G = "CGGCT.A.T" ; $CONSENSUS_1H = "CGGCTA..T" ; $CONSENSUS_1I = "CGGCTAA.." ;
$CONSENSUS_2A = "T.ACAAAAC" ;$CONSENSUS_2B = "TG.CTAAAC" ;$CONSENSUS_2C = "TGA.TAAAC" ;
$CONSENSUS_2D = "TGAC.AAAC" ;$CONSENSUS_2E = "TGACT.AAC" ;$CONSENSUS_2F = "TGACTA.AC" ;
$CONSENSUS_2G = "TGACTAA.C" ;$CONSENSUS_2H = "TGACTAAA." ;$CONSENSUS_2I = ".GACTAAAC" ;
$CONSENSUS_3A = ".GTTTGATC" ;$CONSENSUS_3B = "A.TTTGATC" ;$CONSENSUS_3C = "AG.TTGATC" ;
$CONSENSUS_3D = "AGT.TGATC" ;$CONSENSUS_3E = "AGTT.GATC" ;$CONSENSUS_3F = "AGTTT.ATC" ;
$CONSENSUS_3G = "AGTTTG.TC" ;$CONSENSUS_3H = "AGTTTGA.C" ;$CONSENSUS_3I = "AGTTTGAT." ;
$CONSENSUS_4A = ".TACTAACT" ; $CONSENSUS_4B = "T.ACTAACT" ; $CONSENSUS_4C = "TT.CTAACT" ;
$CONSENSUS_4D = "TTA.TAACT" ; $CONSENSUS_4E = "TTAC.AACT" ; $CONSENSUS_4F = "TTACT.ACT" ;
$CONSENSUS_4G = "TTACTA.CT" ; $CONSENSUS_4H = "TTACTAA.T" ; $CONSENSUS_4I = "TTACTAAC." ;

$LIST_CONSENSUS = "$CONSENSUS_1A,$CONSENSUS_1B,$CONSENSUS_1C,$CONSENSUS_1D,$CONSENSUS_1E" ;
$LIST_CONSENSUS = $LIST_CONSENSUS.=",$CONSENSUS_1F,$CONSENSUS_1G,$CONSENSUS_1H,$CONSENSUS_1I" ;
$LIST_CONSENSUS =
$LIST_CONSENSUS.=",$CONSENSUS_2A,$CONSENSUS_2B,$CONSENSUS_2C,$CONSENSUS_2D,$CONSENSUS_2E" ;
$LIST_CONSENSUS = $LIST_CONSENSUS.=",$CONSENSUS_2F,$CONSENSUS_2G,$CONSENSUS_2H,$CONSENSUS_2I" ;
$LIST_CONSENSUS =
$LIST_CONSENSUS.=",$CONSENSUS_3A,$CONSENSUS_3B,$CONSENSUS_3C,$CONSENSUS_3D,$CONSENSUS_3E" ;
$LIST_CONSENSUS = $LIST_CONSENSUS.=",$CONSENSUS_3F,$CONSENSUS_3G,$CONSENSUS_3H,$CONSENSUS_3I" ;
$LIST_CONSENSUS =
$LIST_CONSENSUS.=",$CONSENSUS_4A,$CONSENSUS_4B,$CONSENSUS_4C,$CONSENSUS_4D,$CONSENSUS_4E" ;
$LIST_CONSENSUS = $LIST_CONSENSUS.=",$CONSENSUS_4F,$CONSENSUS_4G,$CONSENSUS_4H,$CONSENSUS_4I" ;

@ARRAY_LIST = split(',', $LIST_CONSENSUS) ; $NUMBER_CONSENSUS = scalar(@ARRAY_LIST) ;

%HASH_COUNT ; $COUNT_HASH_SET_UP = 0 ;
while ($COUNT_HASH_SET_UP < $NUMBER_CONSENSUS)       {
        $HASH_COUNT{$COUNT_HASH_SET_UP} = 0 ;
        $COUNT_HASH_SET_UP = $COUNT_HASH_SET_UP + 1 ;
                                                                                        }

$OUTPUT_NOT_FOUND = "" ; $OUTPUT_FOUND = "" ; $OUTPUT_SUMMARY = "" ;
$READ_COUNT = 0 ; $COUNT_NOT_FOUND = 0 ;
while (<PHYLIP>)       {
        $READ_COUNT = $READ_COUNT + 1 ; my $COUNT_THOUSAND = $READ_COUNT/2000 ;
        my $COUNT_INTEGER = int($COUNT_THOUSAND) ; my $INTEGER_CHECK = $COUNT_THOUSAND -
$COUNT_INTEGER;
        if ($INTEGER_CHECK == 0) {print "Checking sequence $READ_COUNT\n";  }

        my $LINE = $_ ;        chomp ($LINE); my @ARRAY_LINE = split('\t',$LINE) ;
        my $NAME = $ARRAY_LINE[0] ;   my $SEQ = $ARRAY_LINE[1] ; my $LENGTH_SEQ = length($SEQ) ;

        my @ARRAY_SEQ_BLOCKS = split('-', $SEQ) ;
        my $NUMBER_OF_BLOCKS = scalar(@ARRAY_SEQ_BLOCKS) ;
        my $LAST_BLOCK = $NUMBER_OF_BLOCKS - 1 ;
        my $LENGTH_FIVE_PRIME = 0 ;
        my $COUNT_BLOCK = 0 ; my $REBUILDING_SEQUENCE = "" ;
        while ($COUNT_BLOCK < $LAST_BLOCK)    {
                my $BLOCK = $ARRAY_SEQ_BLOCKS[$COUNT_BLOCK] ;
                $REBUILDING_SEQUENCE = $REBUILDING_SEQUENCE.="$BLOCK-" ;
                $COUNT_BLOCK = $COUNT_BLOCK + 1 ;

        }
        my $LENGTH_FIVE_PRIME = length($REBUILDING_SEQUENCE) ;
        my $DOWNSTREAM_REGION_TO_SCREEN = $ARRAY_SEQ_BLOCKS[$LAST_BLOCK ] ;
        my $LENGTH_DOWNSTREAM_TO_SCREEN = length($DOWNSTREAM_REGION_TO_SCREEN) ;
        my $COUNT_ARRAY = 0 ; my $CONSENSUS_FOUND = 0 ;
        while ($COUNT_ARRAY < $NUMBER_CONSENSUS and $CONSENSUS_FOUND < 1)   {
                my $CURRENT_CONSENSUS = $ARRAY_LIST[$COUNT_ARRAY] ;
                my $LENGTH_CONSENSUS = length($CURRENT_CONSENSUS) ;
                if ($DOWNSTREAM_REGION_TO_SCREEN =~ /$CURRENT_CONSENSUS/)    {
                        $CONSENSUS_FOUND = 2 ;
                        my $INFO_TO_UPDATE = $HASH_COUNT{$COUNT_ARRAY} ;
```

```
                        $INFO_TO_UPDATE = $INFO_TO_UPDATE + 1 ;
                        $HASH_COUNT{$COUNT_ARRAY} = $INFO_TO_UPDATE ;


                        my $FIVE_PRIME_BOUNDARY =  0 ; my $THREE_PRIME_BOUNDARY = 0 ;
                        my $DOWNSTREAM_LENGTH = 0 ; my $THREE_PRIME_FOUND =  0 ;
                        my $LENGTH_TO_SCREEN = length($CURRENT_CONSENSUS) ;
                        while ($THREE_PRIME_FOUND < 1)        {
                                my $DOWNSTREAM_SEARCH = substr($DOWNSTREAM_REGION_TO_SCREEN, 0,
$LENGTH_TO_SCREEN) ;
                                if ($DOWNSTREAM_SEARCH !~ /$CURRENT_CONSENSUS/)      {
                                        $LENGTH_TO_SCREEN = $LENGTH_TO_SCREEN + 1 ;

                        }
                                if ($DOWNSTREAM_SEARCH =~ /$CURRENT_CONSENSUS/)      {
                                        $FIVE_PRIME_BOUNDARY = $LENGTH_TO_SCREEN -
$LENGTH_CONSENSUS;
                                        $THREE_PRIME_BOUNDARY = $LENGTH_TO_SCREEN ;
                                        $DOWNSTREAM_LENGTH = $LENGTH_DOWNSTREAM_TO_SCREEN -
$THREE_PRIME_BOUNDARY ;
                                        $THREE_PRIME_FOUND =  2 ;

                        }

                                                                                            }
                        my $FIVE_PRIME_REGION = substr($SEQ, 0, $LENGTH_FIVE_PRIME) ;
                        my $CONSERVED_REGION =
substr($DOWNSTREAM_REGION_TO_SCREEN,$FIVE_PRIME_BOUNDARY, $LENGTH_CONSENSUS) ;
#                       my $LENGTH_V3_NOT_ALIGNED = $FIVE_PRIME_BOUNDARY - $LENGTH_FIVE_PRIME ;
                        my $LENGTH_V3_NOT_ALIGNED = $FIVE_PRIME_BOUNDARY ;
                        my $V3 = substr($DOWNSTREAM_REGION_TO_SCREEN,0, $LENGTH_V3_NOT_ALIGNED);
                        my $NUMBER_OF_DASHES = $V3_TOTAL_LENGTH - $LENGTH_V3_NOT_ALIGNED ;
                        my $DASHES_COUNT = 0 ; my $V3_DASHES = "" ;
                        while ($DASHES_COUNT < $NUMBER_OF_DASHES)     {
                                $V3_DASHES = $V3_DASHES.="-" ;
                                $DASHES_COUNT = $DASHES_COUNT + 1 ;

        }
                        my $DOWNSTREAM = substr($DOWNSTREAM_REGION_TO_SCREEN,$THREE_PRIME_BOUNDARY
,$DOWNSTREAM_LENGTH) ;
                        my $ALIGNED_SEQ = "$FIVE_PRIME_REGION-$V3_DASHES-$V3-$CONSERVED_REGION-
$DOWNSTREAM"  ;
#                       $OUTPUT_FOUND = $OUTPUT_FOUND.="$LINE\n" ;
                        $OUTPUT_FOUND = $OUTPUT_FOUND.="$NAME\t$ALIGNED_SEQ\n" ;

                                }
                $COUNT_ARRAY = $COUNT_ARRAY + 1 ;

                                        }

        if ($CONSENSUS_FOUND < 1)      {
#               $COUNT_NOT_FOUND = $COUNT_NOT_FOUND + 1 ;
                $OUTPUT_NOT_FOUND = $OUTPUT_NOT_FOUND."$LINE\n" ;
                                                                    }
                                }
close (PHYLIP);
####################################
print "Preparing frequency output\n" ;
$COUNT_OUTPUT = 0;
while ($COUNT_OUTPUT < $NUMBER_CONSENSUS)     {
        my $CURRENT_CONSENSUS = $ARRAY_LIST[$COUNT_OUTPUT] ;
        my $FREQUENCY = $HASH_COUNT{$COUNT_OUTPUT} ;
        $OUTPUT_SUMMARY = $OUTPUT_SUMMARY.="Frequency of $CURRENT_CONSENSUS:\t$FREQUENCY\n" ;
        $COUNT_OUTPUT = $COUNT_OUTPUT + 1 ;
                                                                                            }



open (OUTPUT, ">OUTPUT_C4_position_adjusted_$INPUT_FILE_NAME") or die "Can't open $file for
reading" ;
print OUTPUT "$OUTPUT_SUMMARY" ;
```

```
print OUTPUT "$OUTPUT_FOUND";
close (OUTPUT);

open (OUTPUT_2, ">OUTPUT_C4_position_NOT_FOUND_$INPUT_FILE_NAME") or die "Can't open $file for
reading" ;
print OUTPUT_2 "$OUTPUT_NOT_FOUND";
close (OUTPUT_2);
```

```
#####################################       16S C4 trim end sequence trim.pl
$FILE_NAME = " NAME OF INPUT FILE" ;
open (PHYLIP_FILE, "$FILE_NAME") or die "Can't open $file for reading" ;

$SEQUENCE_TO_FIND = "..-.........-.................TAA" ;
$LENGTH_SEQUENCE_TO_FIND = length($SEQUENCE_TO_FIND) ;

print "Screening for nt sequence\n" ;
$READ_COUNT = 0 ;   $OUTPUT_MATCHED = "" ; $OUTPUT_NOT_MATCHED = "" ; $OUTPUT_TRIMMED = "" ;

while (<PHYLIP_FILE>)  {
        $READ_COUNT = $READ_COUNT + 1 ; my $COUNT_THOUSAND = $READ_COUNT/5000 ;
        my $COUNT_INTEGER = int($COUNT_THOUSAND) ; my $INTEGER_CHECK = $COUNT_THOUSAND -
$COUNT_INTEGER;
        if ($INTEGER_CHECK == 0) {print "Processing sequence $READ_COUNT\n";       }

        my $LINE = $_; chomp ($LINE); my @ARRAY_LINE = split('\t',$LINE);
        my $NAME = $ARRAY_LINE[0] ; my $SEQUENCE = $ARRAY_LINE[1] ;

        my $LENGTH_SEQUENCE = length($SEQUENCE) ;
        my $START_OF_END_TO_CHECK = $LENGTH_SEQUENCE - $LENGTH_SEQUENCE_TO_FIND ;
        my $END_TO_CHECK = substr($SEQUENCE,$START_OF_END_TO_CHECK,$LENGTH_SEQUENCE_TO_FIND) ;

        if ($END_TO_CHECK =~ /$SEQUENCE_TO_FIND/)    {
                $OUTPUT_MATCHED = $OUTPUT_MATCHED.="$NAME\t$SEQUENCE\n" ;
                                                                                        }

        if ($END_TO_CHECK !~ /$SEQUENCE_TO_FIND/)    {
                my $SEQUENCE_TO_TRIM_FOUND = 0 ;
                my $START_POSITION_TRIM = $LENGTH_SEQUENCE - $LENGTH_SEQUENCE_TO_FIND ;
                while ($SEQUENCE_TO_TRIM_FOUND < 1 and $START_POSITION_TRIM > 0)    {
                        my $END_TO_TRIM =
substr($SEQUENCE,$START_POSITION_TRIM,$LENGTH_SEQUENCE_TO_FIND) ;
                        my @ARRAY_END_TO_TRIM = split('',$END_TO_TRIM) ;
                        my $COUNTER_END_TO_TRIM_CHECK = 0 ; my $NUMBER_DASHES = 0 ;
                        while ($COUNTER_END_TO_TRIM_CHECK < $LENGTH_SEQUENCE_TO_FIND)       {
                                my $CURRENT_CHARACTER =
$ARRAY_END_TO_TRIM[$COUNTER_END_TO_TRIM_CHECK] ;
                                if ($CURRENT_CHARACTER =~ /\-/) {$NUMBER_DASHES = $NUMBER_DASHES +
1 ;}
                                $COUNTER_END_TO_TRIM_CHECK = $COUNTER_END_TO_TRIM_CHECK + 1 ;

                                                }
                        if ($NUMBER_DASHES < 3) {
                                if ($END_TO_TRIM =~ /$SEQUENCE_TO_FIND/) {
                                my $LENGTH_TRIMMED = $START_POSITION_TRIM +
$LENGTH_SEQUENCE_TO_FIND;
                                my $TRIMMED_SEQ_FOR_OUTPUT = substr($SEQUENCE,0,$LENGTH_TRIMMED) ;
                                $OUTPUT_TRIMMED =
$OUTPUT_TRIMMED.="$NAME\t$TRIMMED_SEQ_FOR_OUTPUT\n" ;
                                $SEQUENCE_TO_TRIM_FOUND = 2 ;
                                                                                        }
                                                                        }
                        $START_POSITION_TRIM = $START_POSITION_TRIM - 1 ;

                                                }
                if ($SEQUENCE_TO_TRIM_FOUND < 1)      {
                        $OUTPUT_NOT_MATCHED = $OUTPUT_NOT_MATCHED.="$NAME\t$SEQUENCE\n" ;
                                                                                }
                                                                                        }
                                                        }
close (PHYLIP_FILE);


print "Writing to output file" ;

open (OUTPUT_1, ">OUTPUT_seqs_matched_to_query_$FILE_NAME") or die "Can't open $file for reading"
;
print OUTPUT_1 "$OUTPUT_MATCHED\n" ;
print OUTPUT_1 "$OUTPUT_TRIMMED" ;
close (OUTPUT_1);
```

```
open (OUTPUT_2, ">OUTPUT_seqs_NOT_matched_to_query_$FILE_NAME") or die "Can't open $file for
reading" ;
print OUTPUT_2 "$OUTPUT_NOT_MATCHED" ;
close (OUTPUT_2);
```

```
######################################        16S alignment length adjustment.pl
$INPUT_FILE_NAME = " NAME OF INPUT FILE" ;
open (PHYLIP, "$INPUT_FILE_NAME") or die "Can't open $file for reading" ;

$EXPECTED_TOTAL_LENGTH = 900  ;
$POSITION_TO_INSERT_DASHES = 710 ;
$OUTPUT_TOO_LONG = "" ; $OUTPUT_OK = "" ; $OUTPUT_ADJUSTED = "" ;
$NUMBER_OK = 0 ; $NUMBER_ADJUSTED = 0 ;
while (<PHYLIP>) {
        $READ_COUNT = $READ_COUNT + 1 ; my $COUNT_THOUSAND = $READ_COUNT/2000 ;
        my $COUNT_INTEGER = int($COUNT_THOUSAND) ; my $INTEGER_CHECK = $COUNT_THOUSAND -
$COUNT_INTEGER;
        if ($INTEGER_CHECK == 0) {print "Checking sequence $READ_COUNT\n";  }

        my $LINE = $_ ;         chomp ($LINE);
        if ($LINE =~ />/) {
                my @ARRAY_LINE = split('\t',$LINE) ;
                my $NAME = $ARRAY_LINE[0] ;   my $SEQ = $ARRAY_LINE[1] ; my $LENGTH_SEQ =
length($SEQ) ;

                if ($LENGTH_SEQ == $EXPECTED_TOTAL_LENGTH)   {
                        $OUTPUT_OK = $OUTPUT_OK.="$NAME\t$SEQ\n" ;
                        $NUMBER_OK = $NUMBER_OK + 1 ;

        }
                if ($LENGTH_SEQ < $EXPECTED_TOTAL_LENGTH)    {
                        my $UPSTREAM = substr($SEQ,0,$POSITION_TO_INSERT_DASHES) ;
                        my $LENGTH_DOWNSTREAM = $LENGTH_SEQ - $POSITION_TO_INSERT_DASHES ;
                        my $DOWNSTREAM =
substr($SEQ,$POSITION_TO_INSERT_DASHES,$LENGTH_DOWNSTREAM) ;
                        my $DASHES_TO_ADD = $EXPECTED_TOTAL_LENGTH - $LENGTH_SEQ ;
                        my $DASHES = "" ;
                        my $COUNT_ADDED_DASHES = 0 ;
                        while ($COUNT_ADDED_DASHES < $DASHES_TO_ADD)         {
                                $DASHES = $DASHES.="-" ;
                                $COUNT_ADDED_DASHES = $COUNT_ADDED_DASHES + 1 ;

                }
                        my $SEQ_ADJUSTED = "$UPSTREAM$DASHES$DOWNSTREAM" ;
                        $OUTPUT_ADJUSTED = $OUTPUT_ADJUSTED.="$NAME\t$SEQ_ADJUSTED\n" ;
                        $NUMBER_ADJUSTED = $NUMBER_ADJUSTED + 1 ;

        }

                if ($LENGTH_SEQ > $EXPECTED_TOTAL_LENGTH)    {
                        $OUTPUT_TOO_LONG = $OUTPUT_TOO_LONG.="$NAME\t$SEQ\n" ;

        }
                                        }
                                }
close (PHYLIP);

open (OUTPUT, ">OUTPUT_adjusted_length_$INPUT_FILE_NAME") or die "Can't open $file for reading" ;
print OUTPUT "$OUTPUT_OK\n" ;
print OUTPUT "$OUTPUT_ADJUSTED\n" ;
close (OUTPUT);

open (OUTPUT_SUMMARY, ">OUTPUT_summary_adjusted_length_$INPUT_FILE_NAME") or die "Can't open
$file for reading" ;
print OUTPUT_SUMMARY "Sequences not needing length adjustment:\t$NUMBER_OK\n" ;
print OUTPUT_SUMMARY "Sequences with adjusted length:\t$NUMBER_ADJUSTED\n" ;
print OUTPUT_SUMMARY "Sequences that are longer than expected:\n" ;
print OUTPUT_SUMMARY "$OUTPUT_TOO_LONG\n" ;
close (OUTPUT_SUMMARY)  ;
```

```
#####################################       OTU clustering_phylip format.pl
NAME OF INPUT FILE
$INPUT_PHYLIP_NAME = " NAME OF INPUT FILE" ;

print "How many OTUs to cluster for:\n" ;
$NUMBER_OF_OTUS = <STDIN>; chomp($NUMBER_OF_OTUS) ;
print "What number would you like to assign to this round of clustering:\n" ;
$OTU_CLUSTERING_ROUND = <STDIN>; chomp($OTU_CLUSTERING_ROUND) ;

$INPUT_CUTOFF = 0.05 ;                       #######ADJUST CUTOFF
$CENTER_CUTOFF = $INPUT_CUTOFF/2 ;

%PHYLIP_HASH ; @ARRAY_NAMES_TO_CLUSTER ;

$READ_COUNT = 0 ; $OUTPUT = "" ; $OUTPUT_COUNTS = "" ;
$OUTPUT_UNCLUSTERED = "" ; $OUTPUT_TIME_LOG = "" ;
print "Loading sequences in hash\n" ;
open (PHYLIP, "$INPUT_PHYLIP_NAME") or die "Can't open $file for reading" ;
while (<PHYLIP>)       {
        $READ_COUNT = $READ_COUNT + 1 ; my $COUNT_THOUSAND = $READ_COUNT/2000 ;
        my $COUNT_INTEGER = int($COUNT_THOUSAND) ; my $INTEGER_CHECK = $COUNT_THOUSAND -
$COUNT_INTEGER;
        if ($INTEGER_CHECK == 0) {print "Loading sequence $READ_COUNT\n";   }

        my $PHYLIP_LINE = $_ ; chomp ($PHYLIP_LINE) ;
        if ($PHYLIP_LINE =~ />/) {
                my @ARRAY_PHYLIP_LINE = split('\t', $PHYLIP_LINE);
                my $NAME = $ARRAY_PHYLIP_LINE[0] ; my $SEQ = $ARRAY_PHYLIP_LINE[1] ;
#CHECK LENGTH OF SEQUENCES
                $PHYLIP_HASH{$NAME} = $SEQ ;
        push (@ARRAY_NAMES_TO_CLUSTER,$NAME) ;
                                                        }
                                        }
close (PHYLIP);

$SIZE_ARRAY_NAMES_TO_CLUSTER = scalar(@ARRAY_NAMES_TO_CLUSTER) ;
$OTU_COUNT = 0 ;
print "Clustering $SIZE_ARRAY_NAMES_TO_CLUSTER sequence reads\n" ;
while ($OTU_COUNT < $NUMBER_OF_OTUS)  {
        print "Reads remaining to cluster: $SIZE_ARRAY_NAMES_TO_CLUSTER\n" ;
        $OTU_COUNT = $OTU_COUNT + 1 ;
        print "Clustering for OTU from cycle $OTU_COUNT\n" ;
        $OUTPUT = $OUTPUT.="\nOTU from cycle $OTU_COUNT\n";
        my $NAME_CENTER_OF_CLUSTER = $ARRAY_NAMES_TO_CLUSTER[0] ;
        $OUTPUT = $OUTPUT.="$NAME_CENTER_OF_CLUSTER," ;
        my $SEQ_CENTER_OF_CLUSTER = $PHYLIP_HASH{$NAME_CENTER_OF_CLUSTER} ;
        my @ARRAY_NT_CENTER_OF_CLUSTER = split('', $SEQ_CENTER_OF_CLUSTER) ;
        my $LENGTH_SEQ = length($SEQ_CENTER_OF_CLUSTER) ;
        my $UNMATCHED_NAMES = "" ;
        my $COUNT_ARRAY_CLUSTER = 1 ;
        while ($COUNT_ARRAY_CLUSTER < $SIZE_ARRAY_NAMES_TO_CLUSTER) {
                my $NAME_NEXT_SEQ = $ARRAY_NAMES_TO_CLUSTER[$COUNT_ARRAY_CLUSTER] ;
                my $NEXT_SEQ = $PHYLIP_HASH{$NAME_NEXT_SEQ} ;
                my @ARRAY_NT_NEXT_SEQ = split('', $NEXT_SEQ) ;
                my $NT_POSITION = 0 ; my $DISSIMILARITY_COUNT = 0 ;
                my $PERCENT_DISSIMILARITY = 0 ;
                while ($NT_POSITION < $LENGTH_SEQ and $PERCENT_DISSIMILARITY <= $CENTER_CUTOFF)
        {
                        my $CURRENT_NT_CENTER = $ARRAY_NT_CENTER_OF_CLUSTER[$NT_POSITION] ;
                        my $CURRENT_NT_NEXT_SEQ       = $ARRAY_NT_NEXT_SEQ[$NT_POSITION] ;
                        if      ($CURRENT_NT_CENTER ne $CURRENT_NT_NEXT_SEQ) {
                                $DISSIMILARITY_COUNT= $DISSIMILARITY_COUNT + 1 ;
                                $PERCENT_DISSIMILARITY = $DISSIMILARITY_COUNT/$LENGTH_SEQ ;

                        }
                        $NT_POSITION = $NT_POSITION + 1;

                                                                                }
                if ($PERCENT_DISSIMILARITY <= $CENTER_CUTOFF)        {
                        $OUTPUT = $OUTPUT.="$NAME_NEXT_SEQ," ;
```

```
        }
                if ($PERCENT_DISSIMILARITY > $CENTER_CUTOFF)         {
                        $UNMATCHED_NAMES = $UNMATCHED_NAMES.="$NAME_NEXT_SEQ\t" ;

        }
                $COUNT_ARRAY_CLUSTER = $COUNT_ARRAY_CLUSTER + 1;

                        }
        chop ($OUTPUT) ;
        @ARRAY_NAMES_TO_CLUSTER = split('\t', $UNMATCHED_NAMES) ;
        $SIZE_ARRAY_NAMES_TO_CLUSTER = scalar(@ARRAY_NAMES_TO_CLUSTER) ;
        my @timeData = localtime(time) ;
        my $YEAR = $timeData[5] + 1900 ;
        my $MONTH = $timeData[4] + 1 ;
        my $DATE_TIME = "$MONTH-$timeData[3]-$YEAR\t$timeData[2]:$timeData[1]";
        $OUTPUT_TIME_LOG = $OUTPUT_TIME_LOG.="OTU $OTU_COUNT done at $DATE_TIME\n" ;
                                                                                }

print "Generating counts information\n" ;
@ARRAY_OUTPUT = split('\n',$OUTPUT) ;
$NUMBER_OTU_LINES = scalar(@ARRAY_OUTPUT) ;
$COUNTS_FOR_COUNTS = 0 ;
while ($COUNTS_FOR_COUNTS < $NUMBER_OTU_LINES)       {
        my $CURRENT_OUTPUT_LINE = $ARRAY_OUTPUT[$COUNTS_FOR_COUNTS] ;
        if ($CURRENT_OUTPUT_LINE !~ />/)                    {
                $OUTPUT_COUNTS = $OUTPUT_COUNTS.="$CURRENT_OUTPUT_LINE\t" ;
                                                                                        }
        if ($CURRENT_OUTPUT_LINE =~ />/)                    {
                my @ARRAY_CURRENT_OUTPUT_LINE = split(',', $CURRENT_OUTPUT_LINE) ;
                my $OTU_COUNT = scalar(@ARRAY_CURRENT_OUTPUT_LINE) ;
                my $OTU_REP = $ARRAY_CURRENT_OUTPUT_LINE[0] ;
                $OUTPUT_COUNTS = $OUTPUT_COUNTS.="$OTU_REP\t$OTU_COUNT\n" ;
                                                                                        }
        $COUNTS_FOR_COUNTS = $COUNTS_FOR_COUNTS + 1 ;
                                                                                        }
print "Creating phylip for unclustered sequences\n" ;
$COUNT_UNCLUSTERED = 0 ;
while ($COUNT_UNCLUSTERED < $SIZE_ARRAY_NAMES_TO_CLUSTER)   {
        my $CURRENT_UNCLUSTERED = $ARRAY_NAMES_TO_CLUSTER[$COUNT_UNCLUSTERED] ;
        my $SEQ_UNCLUSTERED = $PHYLIP_HASH{$CURRENT_UNCLUSTERED} ;
        $OUTPUT_UNCLUSTERED = $OUTPUT_UNCLUSTERED.="$CURRENT_UNCLUSTERED\t$SEQ_UNCLUSTERED\n" ;
        $COUNT_UNCLUSTERED = $COUNT_UNCLUSTERED + 1 ;

                }

open (OUTPUT,
">OUTPUT_$INPUT_PHYLIP_NAME\_cluster_$INPUT_CUTOFF\_round_$OTU_CLUSTERING_ROUND.list") or die
"Can't open $file for reading" ;
print OUTPUT "$OUTPUT";
close (OUTPUT);
open (OUTPUT2,
">OUTPUT_$INPUT_PHYLIP_NAME\_cluster_$INPUT_CUTOFF\_round_$OTU_CLUSTERING_ROUND.count") or die
"Can't open $file for reading" ;
print OUTPUT2 "$OUTPUT_COUNTS";
close (OUTPUT2);
open (OUTPUT3,
">OUTPUT_$INPUT_PHYLIP_NAME\_unclustered_reads\_round_$OTU_CLUSTERING_ROUND.phylip") or die
"Can't open $file for reading" ;
print OUTPUT3 "$OUTPUT_UNCLUSTERED";
close (OUTPUT3);
open (OUTPUT4,
">OUTPUT_$INPUT_PHYLIP_NAME\_cluster_$INPUT_CUTOFF\_time_log\_round_$OTU_CLUSTERING_ROUND.txt")
or die "Can't open $file for reading" ;
print OUTPUT4 "$OUTPUT_TIME_LOG";
close (OUTPUT4);
```
